# Chemically Tuning Resveratrol for the Effective Killing of Gram-Positive Pathogens

**DOI:** 10.1021/acs.jnatprod.1c01107

**Published:** 2022-05-27

**Authors:** Rubén Cebrián, Qian Li, Pablo Peñalver, Efres Belmonte-Reche, María Andrés-Bilbao, Ricardo Lucas, María Violante de Paz, Oscar P. Kuipers, Juan Carlos Morales

**Affiliations:** † Department of Molecular Genetics, Groningen Biomolecular Sciences and Biotechnology Institute, University of Groningen, Nijenborgh 7, 9747AG Groningen, The Netherlands; ‡ Department of Biochemistry and Molecular Pharmacology and Instituto de Parasitología y Biomedicina López Neyra, CSIC, PTS Granada, Avenida del Conocimiento, 17, 18016 Armilla, Granada, Spain; § Department of Organic and Pharmaceutical Chemistry, School of Pharmacy, University of Seville, 41012 Seville, Spain

## Abstract

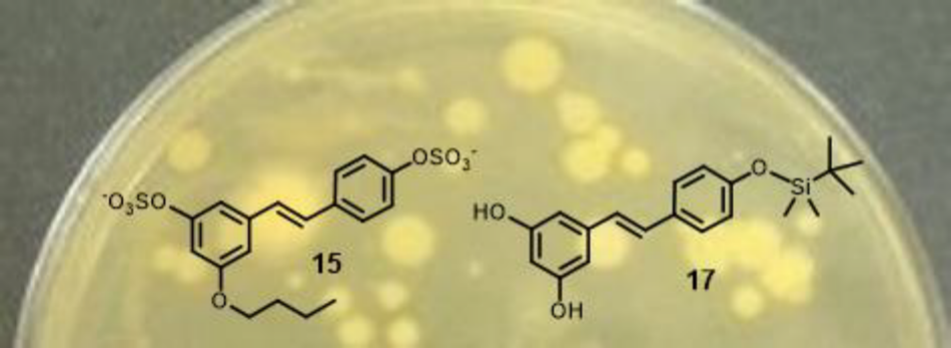

In the era of antimicrobial resistance, the identification of new compounds with strong antimicrobial activity and the development of alternative therapies to fight drug-resistant bacteria are urgently needed. Here, we have used resveratrol, a safe and well-known plant-derived stilbene with poor antimicrobial properties, as a scaffold to design several new families of antimicrobials by adding different chemical entities at specific positions. We have characterized the mode of action of the most active compounds prepared and have examined their synergistic antibacterial activity in combination with traditional antibiotics. Some alkyl- and silyl-resveratrol derivatives show bactericidal activity against Gram-positive bacteria in the same low micromolar range of traditional antibiotics, with an original mechanism of action that combines membrane permeability activity with ionophore-related activities. No cross-resistance or antagonistic effect was observed with traditional antibiotics. Synergism was observed for some specific general-use antibiotics, such as aminoglycosides and cationic antimicrobial peptide antibiotics. No hemolytic activity was observed at the active concentrations or above, although some low toxicity against an MRC-5 cell line was noted.

The increasing number of antimicrobial resistance occurrences together with the decrease in the number of antimicrobial agents approved for human usage constitutes one of the most challenging problems for global health. Thus, new drugs with enhanced antimicrobial activities against multidrug-resistant bacteria (MDR) as well as new therapeutic targets and/or new mechanisms of action are urgently needed.^1,2^ However, and despite the effort being made by the WHO and the research community, the number of new antibiotic drugs approved during the last decades fulfilling these characteristics is low. These newly approved drugs are very often variants of already known antibiotics, offering only a temporary therapeutic solution, since the mechanisms of resistance against them are already established in the pangenome of the bacterial populations.^3,4^

Natural phenolic compounds have been proposed as potential templates to develop new antimicrobials.^5,6^ These chemically diverse molecules are part of the natural plant defense systems against pathogens and could provide an extraordinary source of new antimicrobials because of their variability and the possibilities to design and prepare new chemical derivatives.^7−10^ Moreover, these phenolic compounds could also be used as adjuvants in synergy with traditional antibiotics enhancing their activity against pathogenic bacteria or even sensitizing the bacteria to thus far inactive antibiotics.^11^ In fact, drug combinations are currently exploited as one of the most promising strategies to extend the life of antibiotics in the antimicrobial resistance era.^12,13^

Resveratrol (RES, **1**), a natural phenolic stilbene, is a well-known molecule with potential as a therapeutic agent in several clinical applications such as anticancer, neuroprotector, cardioprotector, and anti-inflammatory agent or for controlling glycemic levels in diabetes or the treatment of nonalcoholic fatty liver disease.^14−16^ Recently, it has also been investigated as an antimicrobial agent.^17,18^ Its activity against bacteria is quite limited, with MIC values in the mM range.^17,19,20^ Synergism of resveratrol with some traditional antibiotics such as aminoglycosides has also been reported. However, the observed synergism is weak and does not result in the complete eradication of pathogenic bacteria.^20,21^ The role of resveratrol in combination with other antibiotics is still controversial, since antagonistic activity or bacterial growth promotion has been observed in some cases.^22,23^ Information about the mechanism of action of resveratrol against bacteria is quite scarce, but it has been related to cell growth inhibition due to suppression of FtsZ expression or ATP synthase activity inhibition.^21,24^ In the case of other natural related stilbene derivatives with a varied number of OH groups, such as pinosylvin and piceatannol,^25,26^ or with methylated groups, such as pinostilbene (PIN) and pterostilbene (PTER),^26−28^ the observed antimicrobial activity was higher than for RES alone or RES in combination with traditional drugs. Moreover, PTER is capable of enhancing the antibiotic activity of known drugs such as gentamicin or polymixin B.^29,30^ Several RES derivatives including methylated^31^ and halogenated/acetylated RES derivatives,^32^ hybrids of RES and pterostilbene,^33,34^ and RES analogues with a furane ring substituting one of the phenol rings of RES^35^ or modifications of the central alkene bond to a triazolyl ring^36^ have been reported to improve RES antimicrobial activity. These data suggest that RES is a versatile scaffold to design new families of antimicrobial drugs.

In this work, we describe the antibacterial activity of several RES derivatives against a broad panel of Gram-negative and Gram-positive pathogens. The modifications we have examined on RES include glucosyl-acyl, alkyl, alkyl-sulfate, silyl, and silyl-acyl derivatives together with two RES metabolites. We have also evaluated the antibacterial activity of derivatives of PIN and PTER modified with glucosyl-acyl and glucuronosyl groups. After a first screening, we synthesized and assayed several newly designed silyl sulfate RES derivatives and silyl RES derivatives, which had an unprecedented potent activity. Finally, we investigated the mechanism of action of the RES derivatives with the best antibacterial activity.
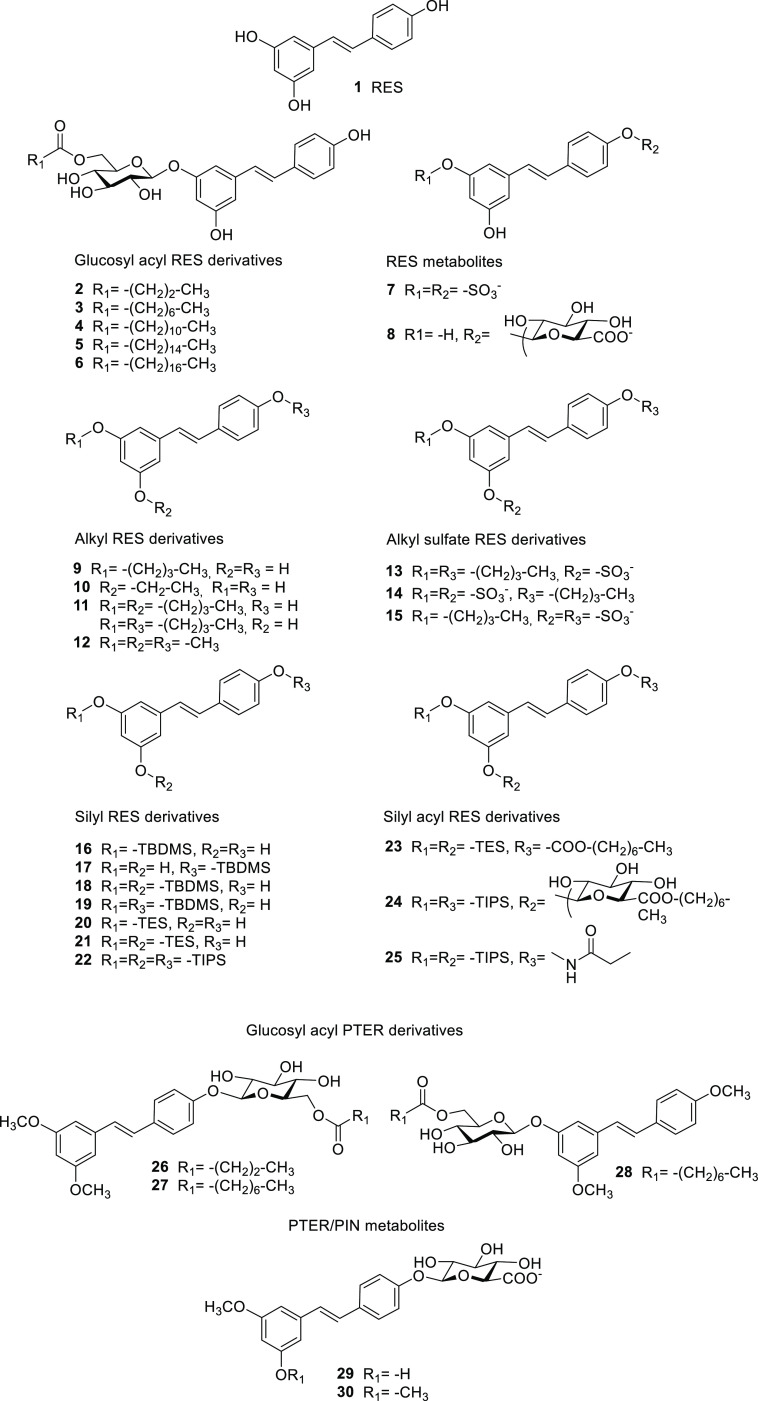


## Results and Discussion

### Preparation of RES Derivatives

A variety of compounds used or designed for noninfectious diseases have shown later to display *in vitro* antimicrobial activity against pathogenic bacteria.^37−39^ On the basis of that finding, we tested a miscellaneous group of RES, PIN, and PTER derivatives (**1**–**30**), previously designed to treat noncommunicable pathologies, against a panel of Gram-negative and Gram-positive bacteria.^40−43^ The examined compounds incorporate either one chemical group in the hydroxyl groups of the stilbene scaffold (glucosyl, acyl, alkyl, sulfate, glucuronate, or silyl) or a combination of two or three of these groups.

Glucosyl-acyl RES derivatives **2**–**6** were prepared from piceid (3-*O*-glucosyl-resveratrol) by enzymatic acylation following the synthetic procedure previously reported.^40^ Resveratrol 3,4′-disulfate (**7**) was synthesized from RES by partial TBDMS protection, sulfation with SO_3_·NMe_3_ and Et_3_N under microwave irradiation, and final silyl deprotection using KF^44^ and purified as previously described.^41^ Resveratrol-3-glucuronate (**8**) was prepared as reported previously.^45^ Briefly, 3,4′-diTBDMS-resveratrol was reacted with a pivaloyl-protected glucuronate trichloroacetimidate donor followed by silyl deprotection using HF·pyridine in tetrahydrofuran (THF) and final acyl deprotection with Na_2_CO_3_ in MeOH/H_2_O. PIN and PTER glucuronates **29** and **30** were similarly synthesized.^46^ Methyl, ethyl, and butyl resveratrol derivatives **9**–**12** were also prepared.^47,48^ Random alkylation of RES was carried out using K_2_CO_3_ and the corresponding 1-iodoalkane, followed by chromatographic separation of the mono-, di-, and trialkyl resveratrol derivatives. Butyl sulfate RES derivatives **13**–**15** were prepared from the corresponding butyl RES derivatives by sulfation with SO_3_·NMe_3_ and Et_3_N under microwave irradiation.^42^ Glucosyl acyl PTER derivatives **26** and **27** as well as the regioisomer **28** were synthesized as previously described.^42^ 3,4′-Dimethyl resveratrol, obtained by random methylation of RES, was glycosylated with peracetylated glucosyl trichloroacetimidate donor and BF_3_·Et_2_O as a catalyst. The corresponding product was deprotected with NaOMe in MeOH and acylated at the primary hydroxyl group using Novozym 435 and vinyl octanoate to yield compound **28**. The same synthetic strategy was applied to PTER to prepare compounds **26** and **27**.

Finally, silyl RES derivatives **16**–**22** were synthesized from RES by random silylation using the corresponding silyl chloride and imidazole in THF followed by separation of the different products using silica gel column chromatography.^43^ The silyl acyl RES derivative **23** was synthesized by direct enzymatic acylation of 3,5-ditriethylsilyl resveratrol using Novozym 435 and vinyl octanoate as previously reported.^41^ The synthesis of compound **24** followed a similar strategy to that used to prepare compounds **26**–**28**. Glycosylation of 3,4′-ditriisopropylsilyl resveratrol with peracetylated glucosyl trichloroacetimidate donor and BF_3_·Et_2_O as catalyst, followed by selective acylation using Novozym 435 and vinyl octanoate, yielded compound **24**.^41^ Silyl ethyl carbamate RES derivative **25** was synthesized by reacting 3,5-ditriisopropylsilyl resveratrol with ethyl isocyanate and Et_3_N.^41^

A stability study was carried out on the four more relevant compounds (**15**, **17**, **32**, and **33**) together with resveratrol (**1**) as control. The incubation was done in the bacterial Muller-Hinton broth (M-H broth) for 24 h and in the Dulbecco’s modified Eagle medium (DMEM) cell media for 72 h at 5 μM concentration of each compound. The results indicate that all compounds were much more stable in DMEM than in M-H broth, with percentages of compound remaining in DMEM higher than in M-H broth in all cases, even though the incubation time in DMEM is longer (Supporting Figure 1). Additionally, it can be observed that the stability of silyl RES derivatives (**17** and **32**), in both DMEM and M-H broth, was less than RES itself and the alkyl-sulfated-RES derivative (**15**). In any case, a minimum of 70% of compound remaining in DMEM and 40% in M-H broth is detectable at the final incubation time of each bioassay.

### Antimicrobial Activity of the First Library of RES Derivatives

No antibacterial activity was observed against Gram-negative bacteria for the RES, PIN, or PTER derivatives in the range examined (128 to 2 μM). On the other hand, several RES and PTER derivatives were active in the low micromolar range against Gram-positive bacteria (Table 1). Among the glucosyl-acyl RES derivatives (also named piceid acylated derivatives), compound **4** containing a medium-size dodecanoyl chain showed good antibacterial activity, especially against *Bacillus* and *Staphylococcus* strains (8 or 16 μM). Interestingly, the activity decreased with smaller acyl chains (compounds **2** and **3**) and also with larger acyl chains (compounds **5** and **6**), although piceid stearate **6** showed some activity against *Enterococcus* strains, especially strong against *E. faecium*. This family of piceid acylated derivatives has been reported to inhibit the adhesion of *Escherichia coli* O157:H7, *Salmonella typhimurium*, and *Listeria monocytogenes* Scott A to Caco-2 and HT-29 colonic cells by 60%, 40%, and 20%, respectively,^49^ although no direct antimicrobial activity was indicated. When glucosyl-acyl modifications were introduced in PIN, PTER, or 3,4′-dimethyl resveratrol (compounds **26**–**28**), only compound **28** showed weak antibacterial activity.

**Table 1:** Minimal Inhibition Concentration (MIC) Observed for the Different RES Derivatives against a Panel of Aerobic and Anaerobic Gram-Positive Bacteria^a^

compound	chemical modification	*B. cereus* ATCC10987	*B. cereus* ATCC14579	*E. faecalis* LMG08222	*E. faecalis* LMG16216	*E. faecalis* V583	*E. faecium* LMG11423	*E. faecium* LMG16003	*S.aureus* LMG8224
**3**	glucosyl-acyl RES	128 ± 0	128 ± 0	128 ± 0	128 ± 0	128 ± 0	128 ± 0	128 ± 0	128 ± 0
**4**		8 ± 0	8 ± 0	32 ± 0	64 ± 0	53.3 ± 10.6	53.3 ± 10.6	53.3 ± 10.6	16 ± 0
**6**		-	-	16 ± 0	128 ± 0	128 ± 0	1 ± 0	2.6 ± 0.6	-
**9**		32 ± 0	32 ± 0	128 ± 0	128 ± 0	128 ± 0	64 ± 0	128 ± 0	64 ± 0
**10**	alkyl RES	128 ± 0	128 ± 0	-	-	-	-	-	-
**11**		64 ± 0	26.6 ± 5.3	21.3 ± 5.3	16 ± 0	42.6 ± 10.6	26.6 ± 5.3	10.6 ± 2.6	-
**14**	alkyl sulfate RES	128 ± 0	128 ± 0	32 ± 0	16 ± 0	64 ± 0	32 ± 0	64 ± 0	32 ± 0
**15**		8 ± 0	6.6 ± 1.3	16 ± 0	16 ± 0	26.6 ± 5.3	32 ± 0	32 ± 0	16 ± 0
**16**	silyl RES	16 ± 0	16 ± 0	8 ± 0	16 ± 0	16 ± 0	16 ± 0	16 ± 0	16 ± 0
**17**		8 ± 0	4 ± 0	6.6 ± 1.3	8 ± 0	8 ± 0	8 ± 0	8 ± 0	8 ± 0
**19**		-	-	-	-	-	-	-	-
**28**	Glc-acyl PTER	128 ± 0	128 ± 0	-	-	-	-	-	128 ± 0
**30**	GlcAc Pter	16 ± 0	13.3 ± 2.6	64 ± 0	128 ± 0	128 ± 0	-	-	32 ± 0
	vancomycin	6 ± 2	nd	nd	nd	9.8 ± 2.7	nd	>32	10.7 ± 2.7
	ciprofloxacin	10.7 ± 2.7	nd	nd	nd	26.7 ± 5.3	nd	32	20.8 ± 4.8
	gentamicin	4 ± 0	nd	nd	nd	>32	nd	12.57 ± 1.61	1 ± 0.35

compound	chemical modification	*S.aureus* LMG10147	*S.aureus* LMG15975	*C. beijerinckii* B504	*C. botulinum* CECT551	*C. difficile* CECT531	*C. ihumii* AP5	*C. perfringens* CECT376	*C. tetani* CECT4629
**3**	glucosyl-acyl RES	128 ± 0	128 ± 0	32 ± 0	32 ± 0	64 ± 0	64 ± 0	128 ± 0	21.3 ± 5.3
**4**		16 ± 0	16 ± 0	16 ± 0	8 ± 0	128 ± 0	32 ± 0	32 ± 0	-
**6**		-	-	-	-	-	-	-	-
**9**		64 ± 0	32 ± 0	26.6 ± 5.3	32 ± 0	64 ± 0	64 ± 0	64 ± 0	16 ± 0
**10**	alkyl RES	-	-	-	-	-	-	-	-
**11**		128 ± 0	-	42.6 ± 10.6	32 ± 0	53.3 ± 10.6	32 ± 0	85.3 ± 21.3	8 ± 0
**14**	alkyl sulfate RES	32 ± 0	32 ± 0	16 ± 0	21.3 ± 5.3	32 ± 0	32 ± 0	32 ± 0	16 ± 0
**15**		8 ± 0	10.6 ± 2.6	32 ± 0	32 ± 0	64 ± 0	32 ± 0	26.6 ± 5.3	16 ± 0
**16**	silyl RES	16 ± 0	16 ± 0	26.6 ± 5.3	16	26.6 ± 5.3	32	32	6.6 ± 1.3
**17**		8 ± 0	8 ± 0	10.6 ± 2.6	13.3 ± 2.6	21.3 ± 5.3	16	13.3 ± 2.6	5.3 ± 1.3
**19**		-	-	32 ± 0	53.3 ± 10.6	-	128 ± 0	128 ± 0	5.3 ± 1.3
**28**	Glc-acyl PTER	128 ± 0	128 ± 0	32 ± 0	26.6 ± 5.3	85.3 ± 21.3	64 ± 0	128 ± 0	16 ± 0
**30**	GlcAc Pter	32 ± 0	32 ± 0	10.7 ± 2.6	4 ± 0	13.3 ± 2.6	8 ± 0	5.3 ± 1.3	4 ± 0
	vancomycin	nd	nd	nd	nd	1.25 ± 0.75	nd	0.18 ± 0.06	nd
	ciprofloxacin	nd	nd	nd	nd	>32	nd	0.5 ± 0	nd
	gentamicin	nd	nd	nd	nd	>32	nd	>32	nd

a The concentrations are expressed in μM. Compounds **1**, **2**, **5**, **7**, **8**, **12**, **13**, **18**, **20**–**27**, and **29** are not shown since they were not active under the conditions examined. - means not active under the conditions examined. nd means not determined.

The RES, PTER, and PIN metabolites **7**, **8**, **29**, and **30** did not show antibiotic activity except PTER glucuronate **30** against *Bacillus cereus*, *Clostridium* strains, and *Clostridioides difficile* with MIC values below 16 μM.

Among the alkyl RES derivatives, the butyl RES compounds **9** and **11** displayed the best MIC values. The 1:1 mixture of 3,5-dibutyl and 3,4′-dibutyl resveratrol compounds **11** resulted in the range between 8 μM for *E. faecium* and *C. tetani* to 128 μM for some *Staphylococcus*. The 3-butyl resveratrol **9** also showed antibacterial activity but to a lesser extent than the dibutyl resveratrol mixture **11**. Interestingly, when the hydroxyl groups were replaced with sulfate groups (compounds **13**–**15**), important changes in antibacterial activity were observed. In fact, 3,4′-dibutyl-5-sulfate resveratrol **13** did not exhibit any antibiotic activity, whereas monobutyl disulfate resveratrol derivatives **14** and **15** were active against all Gram-positive bacterial strains examined. At the same time, the position where the sulfates are located is also relevant. In fact, 3-butyl-4′,5-disulfate resveratrol **15** showed MIC values of 8 μM against *Bacillus* and *Staphylococcus* strains and 16 μM against *Enterococcus* and *Clostridium* strains. However, the regioisomer 4′-butyl-3,5-disulfate resveratrol **14** exhibited higher MIC values for most bacterial strains than those obtained for compound **15**.

According to Table 1, the compounds with the best antibacterial activity in this first screening were among the family of silyl RES derivatives (**16**–**22**). 3-*tert*-Butyldimethylsilyl resveratrol **16** and 4′-*tert*-butyldimethylsilyl resveratrol **17** exhibited MIC values ranging from 4 to 21 μM and from 8 to 32 μM, respectively, against Gram-positive bacterial strains examined. In contrast, another monosilyl RES derivative, the 3-triethylsilyl resveratrol **20**, showed no activity, which may be due to the potential lability of these silyl groups in weak acid or basic conditions. At the same time, the disilyl and trisilyl RES derivatives (**18**, **19** and **21**, **22**) were not active or displayed very low antibacterial activity such as compound **19** against *Clostridium* strains. Further modifications on silyl RES derivatives with acyl, glucosyl-acyl, or carbamate (**23**–**25**) did not improve antibacterial activity for any strain examined. It must be added that the activities found for the RES derivatives were much better than those described previously for RES alone, which are usually in the mM or high μM range.^17,19^

### RES Derivative Redesign and Antibacterial Potency

After the first screening, silyl RES derivatives **16** and **17** together with 3-butyl-4′,5-disulfate resveratrol **15** showed the best antibacterial activity. Based on these results, a second set of compounds was prepared to try to improve their bioactivity. Thus, another three monosilyl RES derivatives (**31**–**33**), as well as new silyl sulfate RES derivatives combining the two best chemical groups able to increase RES antibiotic activity, sulfates and silyl groups (**34**–**36**), were synthesized.

The synthesis of monosilyl RES derivatives **31**–**33** was carried out following the procedure described above. Silyl sulfate RES derivatives **34**–**36** were synthesized by sulfation of the corresponding TBDMS or TIPS RES derivatives with SO_3_·NMe_3_ and Et_3_N under microwave irradiation followed by silica gel column chromatography purification in hexane/EtOAc mixtures.
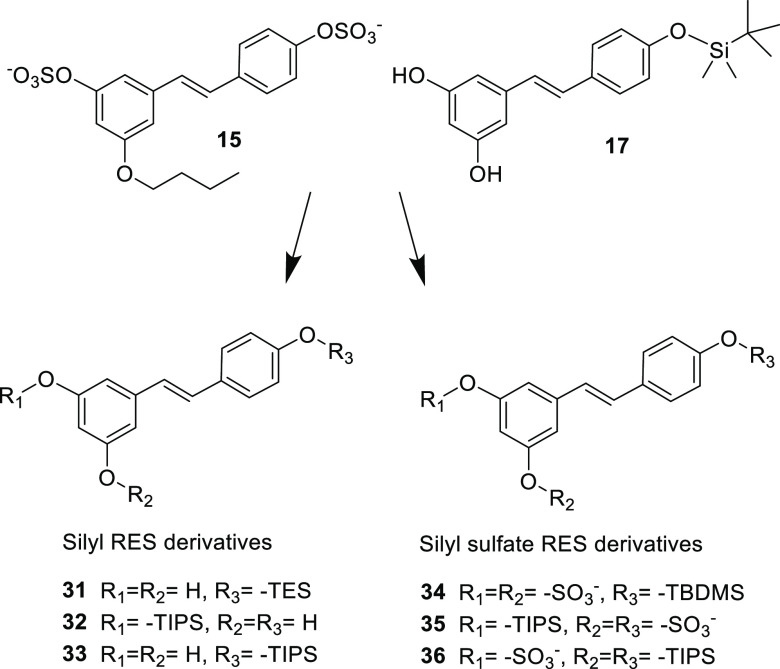


The RES derivative containing the triethylsilyl group at a new position in the stilbene scaffold (**31**) was not active, pointing to the potential reasoning about its lability (Table 2). In contrast, tri-isopropylsilyl monosubstituted RES derivatives **32** and **33** resulted in quite active compounds, with MIC values for **32** from 4 to 8 μM against *Bacillus, Enterococcus*, and *Staphylococcus* strains and from 8 to 16 μM against the Gram-positive anaerobic strains.

**Table 2:** Minimal Inhibition Concentration (MIC) Observed for the Different RES Derivatives against a Panel of Aerobic and Anaerobic Gram-Positive Bacteria^a^

compound	Chemical modification	*B. cereus* ATCC10987	*B. cereus* ATCC14579	*E. faecalis* LMG08222	*E. faecalis* LMG16216	*E. faecalis* V583	*E. faecium* LMG11423	*E. faecium* LMG16003	*S.aureus* LMG8224
**31**	silyl	-	-	-	-	-	-	-	-
**32**		8 ± 0	8 ± 0	4 ± 0	4 ± 0	8 ± 0	4 ± 0	4 ± 0	8 ± 0
**33**		32	26.6 ± 5.3	8 ± 0	8 ± 0	21.3 ± 5.3	16 ± 0	5.3 ± 1.3	128 ± 0
**34**	silyl sulfate	-	-	-	-	-	-	-	-
**35**		16	21.3 ± 5.3	13.3 ± 2.6	8 ± 0	16 ± 0	16 ± 0	13.3 ± 2.6	13.3 ± 2.6
**36**		-	-	128 ± 0	-	128 ± 0	-	-	-

compound	Chemical modification	*S.aureus* LMG10147	*S.aureus* LMG15975	*C. beijerinckii* B504	*C. botulinum* CECT551	*C. difficile* CECT531	*C. ihumii* AP5	*C. perfringens* CECT376	*C. tetani* CECT4629
**31**	silyl	-	-	-	-	-	-	-	-
**32**		8 ± 0	8 ± 0	10.7 ± 2.6	8 ± 0	16 ± 0	16 ± 0	16 ± 0	5.3 ± 1.3
**33**		128 ± 0	128 ± 0	21.3 ± 5.3	10.7 ± 2.6	16 ± 0	13.3 ± 2.6	26.6 ± 5.3	6.7 ± 1.3
**34**	silyl sulfate	-	-	32 ± 0	53.3 ± 10.6	-	128	128	5.3 ± 1.3
**35**		21.3 ± 05.3	16 ± 0	64 ± 0	64 ± 0	32 ± 0	64 ± 0	64 ± 0	64 ± 0
**36**		-	-	-	-	-	-	-	-

a The concentrations are expressed in μM. - means not active under the conditions examined.

The introduction of sulfates in positions 3 and 5 of compound **17**, resulting in compound **34**, almost abolished the antimicrobial activity. Only Gram-positive anaerobic strains were sensitive to **34**, especially *C. tetani*, which suggests a positional effect of the sulfate groups that could be related to their antibacterial efficacy (Table 2). When sulfates were located at positions 3 and 4′, as in compound **15**, with a tri-isopropylsilyl group instead of a butyl group at position 5 (**35**), we observed antibacterial activity against all examined bacterial strains, with MIC values ranging from 8 to 64 μM. However, testing the silyl sulfate RES derivatives did not improve the antibacterial activity observed for the monosilyl RES derivatives **16**, **17**, **32**, and **33** or for the alkyl sulfate RES derivative **15**. As before, none of the new set of compounds (**31**–**36**) was active against Gram-negative bacteria in a similar way to that observed for RES and PTER derivatives **2**–**30**.

### The Gram-negative Outer Membrane Acts as a Permeability Barrier

Although some of the designed RES derivatives showed strong antimicrobial activity against Gram-positive bacteria (Tables 1 and 2), none were active against Gram-negative pathogens. Infections produced by Gram-negative bacteria represent one of the greatest challenges faced by global health. Their high resistance to a high range of antimicrobials is due, among other reasons, to the presence of the external membrane that acts as a true permeability barrier for these antimicrobials.^50^ For this reason, we investigated if the outer membrane prevents RES derivatives from entering the cells. Thus, we permeabilized the outer membrane of *E. coli* LMG8223 using the outer-membrane-perturbing peptide L-11^51^ and tested eight selected RES derivatives active against Gram-positive bacteria (**4**, **15**, **17**, **30**, **32**, **33**, **35**, and **37**). The MIC values measured for these compounds in the presence and absence of 4 μM of L-11 are listed in Table 3. Permeabilized *E. coli* cells were sensitive to RES derivatives in the range of concentrations found for Gram-positive bacteria. These data suggest that the target(s) for these drugs is(are) also present in Gram-negative bacteria, but the outer membrane prevents these compounds from reaching their targets.

**Table 3:** Antimicrobial Activity of Selected RES Derivatives against *E. coli* LMG8224 in the Presence or Absence of L-11 Outer-Membrane-Perturbing Peptide

	MIC (μM)	
	L-11	
compound	+ 0 μM	+ 4 μM
**4**	>128	6.6 ± 1.3
**15**	>128	10.6 ± 2.6
**17**	>128	13.3 ± 2.6
**30**	>128	16 ± 0
**32**	>128	8 ± 0
**33**	>128	21.3 ± 5.3
**35**	>128	21.6 ± 5.3
**37**	>128	16 ± 0

### Bactericidal/Bacteriostatic Activity of RES Derivatives

**Figure 1:**
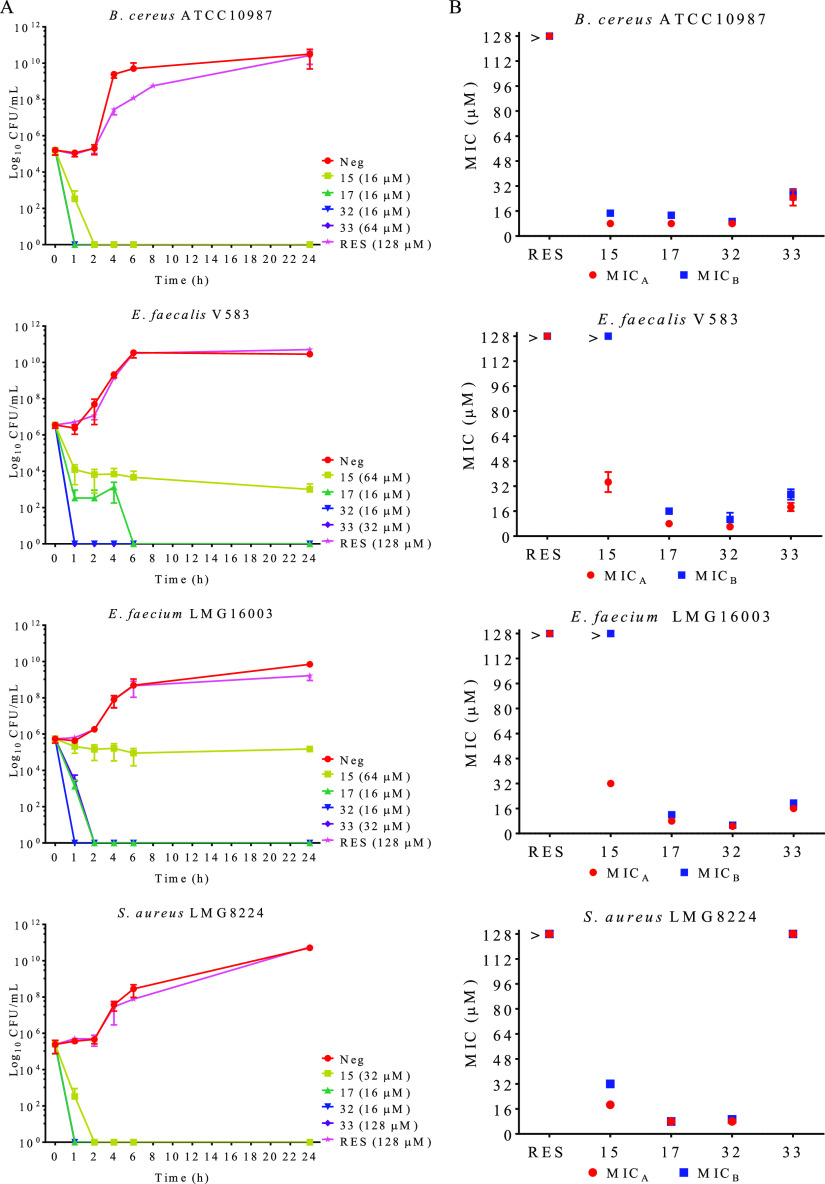
Bactericidal/bacteriostatic effect analysis. (A) Killing kinetics for resveratrol (RES) and the different selected RES derivatives, compounds **15**, **17**, **32**, and **33**, at 2-fold the MIC concentration. The data represent the average of CFU/mL ± SEM. (B) Bactericidal/bacteriostatic activity of RES and the different selected RES derivatives, compounds **15**, **17**, **32**, and **33**. Neg: negative control (nontreated cells). MIC_A_ indicates the MIC obtained for the bacteria after a first MIC test ± SEM. MIC_B_ indicates the minimal bactericidal concentration ± SEM. > indicates that the bacteria grew above this concentration for the tested compounds. All the tests were performed in triplicate.

We selected four compounds among those showing the best antibacterial activity to investigate their mechanism of action. Three of them were monosilyl RES derivatives **17**, **32**, and **33**, and the fourth was 3-butyl-4′,5-disulfate resveratrol **15**. First, we tested the killing kinetics to measure the potential bactericidal or bacteriostatic effect of the selected RES derivatives. *B. cereus* ATCC 10987, *E. faecalis* V583, *E. faecium* LMG 16003, and *S. aureus* LMG 8224 were cultivated in cationic-adjusted Mueller-Hinton broth medium (cMHB) in the presence of 2-fold the MIC during 24 h. At different times, a sample of the culture was withdrawn and decimal-serially diluted, and the CFU/mL was calculated. As indicated in Figure 1A, overall, the compounds showed bactericidal activity in a few hours against the four tested bacteria, except derivative **15**, which was bacteriostatic when *Enterococcus* strains were tested (Figure 1A). To confirm the bactericidal/bacteriostatic effect, the minimal bactericidal concentration was determined. After a first MIC test at different concentrations of the compounds (128 to 2 μM) in a 96-well plate for 20 h, a new cMHB medium was added to the 96-well plate, and it was inoculated at 10% using the previous one as inoculum. Under these conditions, the absence of bacterial growth indicates a bactericidal effect. Unlike resveratrol, for which bacteriostatic activity has been previously reported,^21,52,53^ the antimicrobial activity of these compounds was mainly bactericidal except for compound **15**, which was bacteriostatic for *Enterococcus* strains (Figure 1B). In this case, and although the MIC for this derivative was around 32 μM for these bacteria, the bacteria grew after the treatment at 128 μM (Figure 1B).

### RES Derivatives Act on Bacterial Membranes Inducing Membrane Permeabilization and Hyperpolarization

**Figure 2:**
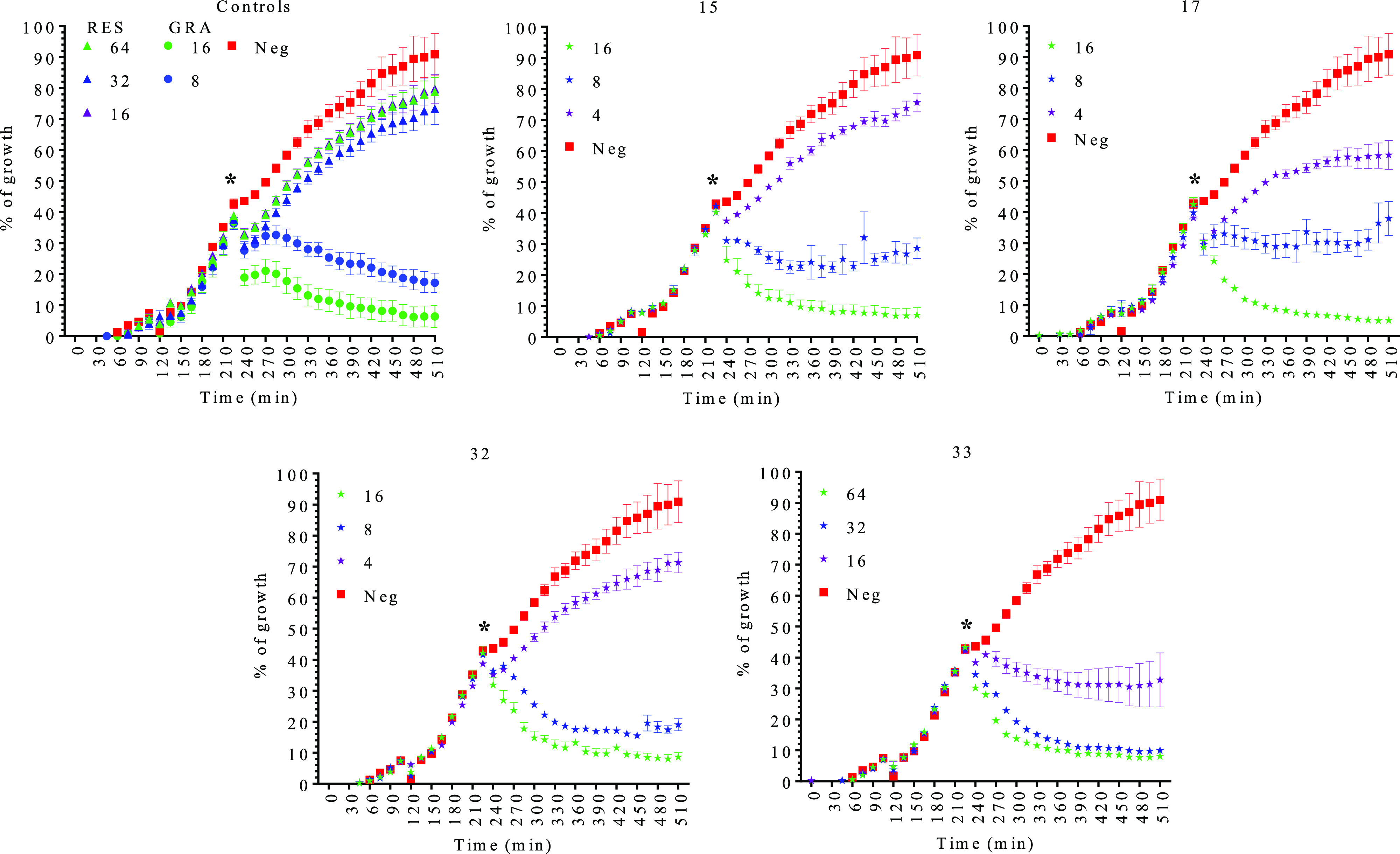
Antimicrobial effect of resveratrol (RES) and the different selected RES derivatives, compounds **15**, **17**, **32**, and **33** on actively growing cells. The % of growth was calculated with respect to the negative control, and it is expressed as the average ± SEM. The concentration of the compounds used is expressed in μM. * indicates compound additions. Gramicidin S (GRA) was used as a positive control. Neg: negative control (nontreated cells).

According to the previous results and considering that RES in high dosage and some others RES derivatives have been previously described as bacterial membrane disruptive agents,^34,54^ we tested if the RES derivatives selected were able to lyse the cells. *B. cereus* ATCC10987 was selected as a model, and compounds at 2×, 1×, and 0.5× the MIC concentration were added at the middle of the exponential growth phase. Subsequently, the OD values were recorded for several hours. Gramicidin S was used as a positive control and resveratrol as a negative control. Figure 2 shows that all the compounds act as lytic compounds in a dose-dependent manner. Compounds **32** and **33** were the most effective derivatives lysing the cells, while compounds **15** and **17** were bacteriostatic at the MIC concentration and bactericidal at 2× the MIC (as observed in Figure 1A and 1B). Overall, the obtained data suggest that the antimicrobial target of the RES derivatives could be the bacterial membrane. Thus, we measured membrane integrity, as well as membrane polarization.

**Figure 3:**
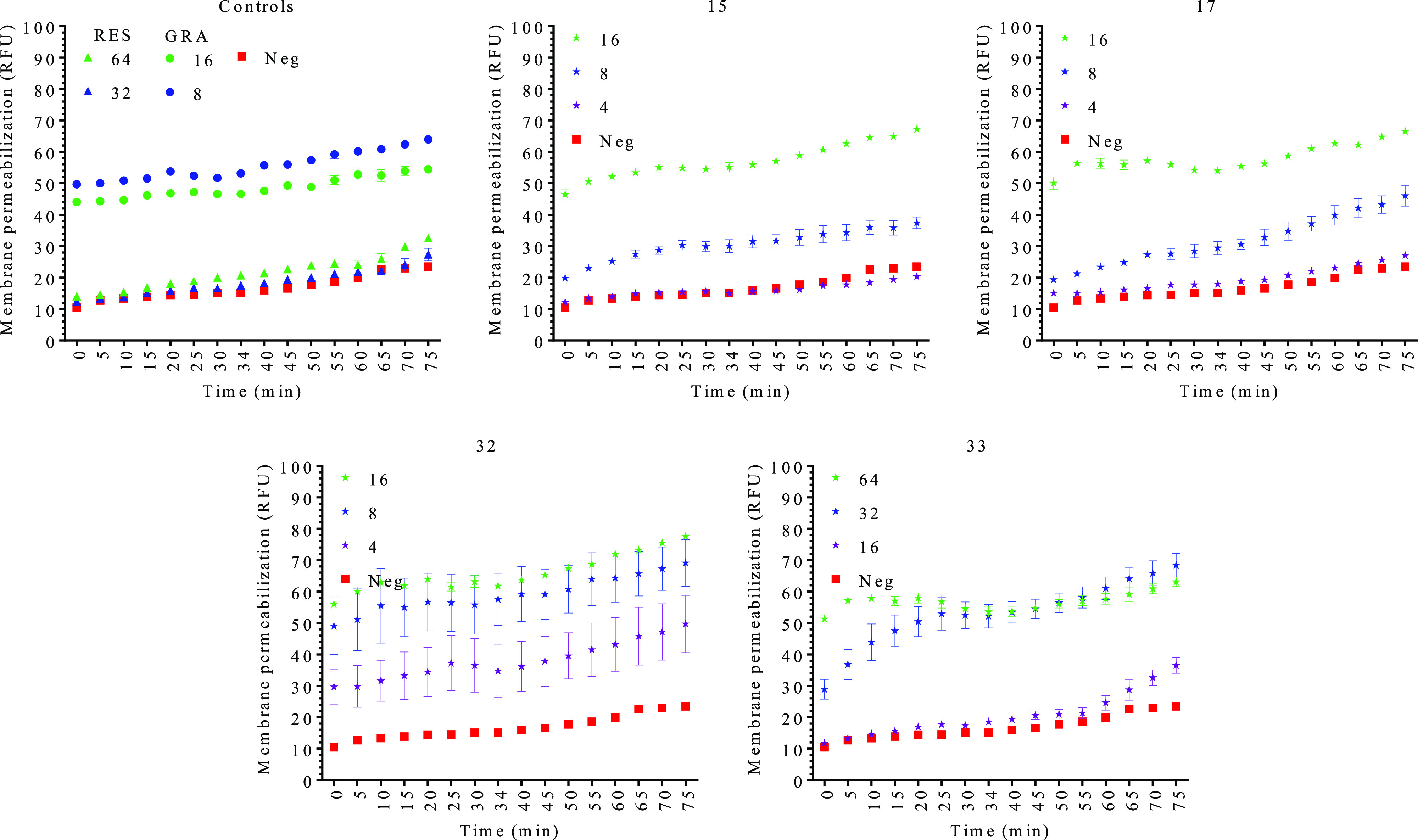
Propidium iodide membrane permeabilization test for resveratrol (RES) and the different selected RES derivatives, compounds **15**, **17**, **32**, and **33**. The data are expressed as the average of random fluorescent units (RFU, fluorescence normalized with the OD_600_) ± SEM. The concentration of the compounds is expressed in μM. Gramicidin S (GRA) was used as a positive control. Neg: negative control (nontreated cells).

First, we evaluated the integrity of the membrane using the propidium iodide uptake test. This dye is fluorescent when binding to DNA, being nonfluorescent when is not able to cross the intact bacterial membranes and cannot reach the bacterial DNA.^55^ As can be seen in Figure 3, all selected RES derivatives induced membrane permeabilization in a dose-dependent manner, while RES did not induce any effect on membrane permeability.^21^ These data support the previous observation (Figures 1A,B and 2) and suggest that the mechanism of action of these derivatives could be related to lysis of the cells by membrane permeabilization. Since membrane permeabilization is usually related to membrane depolarization, the membrane potential was also measured using the membrane potential-sensitive dye DiSC3(5).^56^

**Figure 4:**
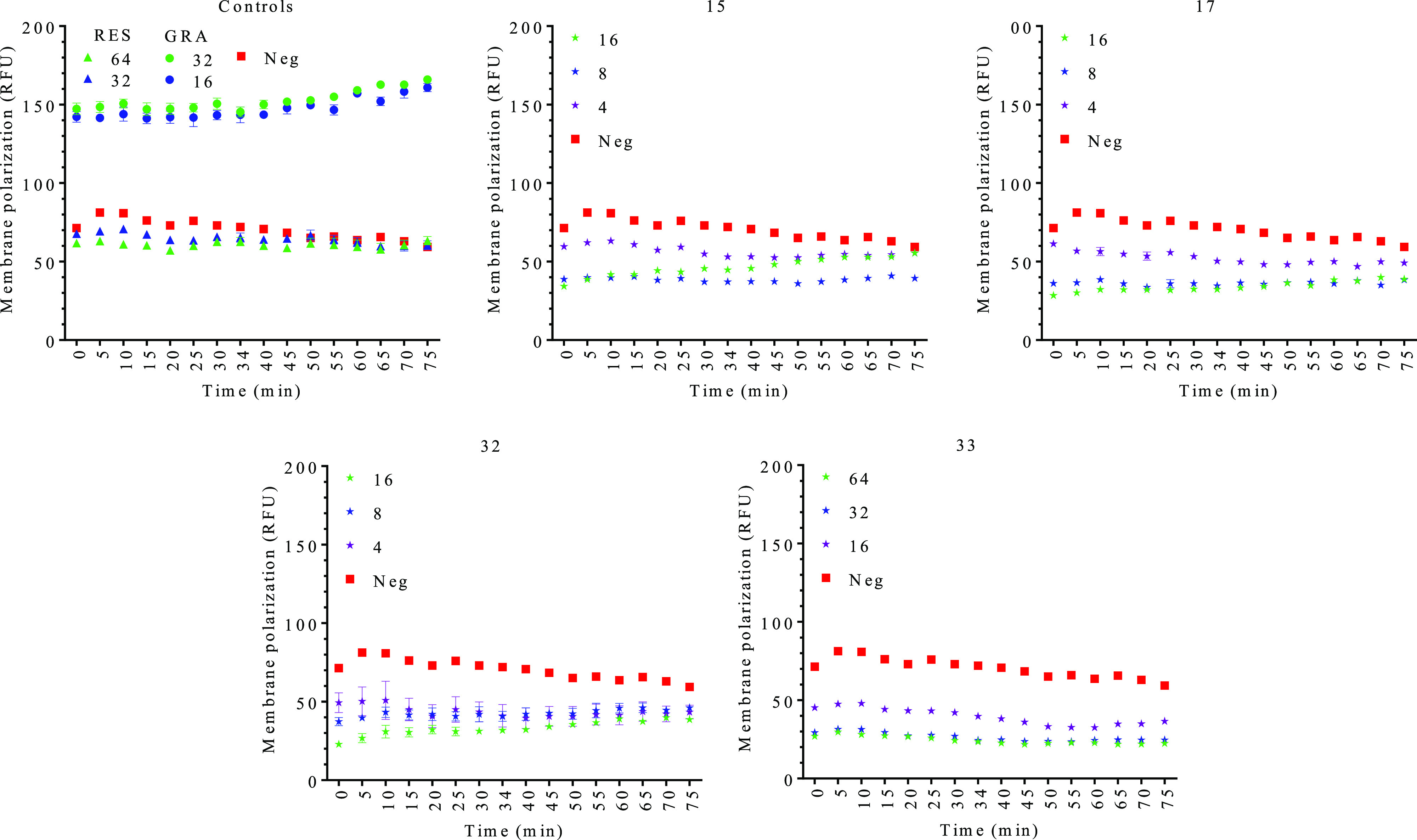
DiSC3(5) membrane potential detection for resveratrol (RES) and the different selected RES derivatives, compounds **15**, **17**, **32**, and **33**. The data are expressed as the average of normalized RFU ± SEM. The concentration of the compounds is expressed in μM. All the tests were performed in triplicate. Gramicidin S (GRA) was used as a positive control. Neg: negative control (nontreated cells).

As shown in Figure 4, RES did not induce any membrane potential change at the tested conditions, while the positive control (gramicidin S) induced a strong membrane depolarization. None of the RES derivatives tested induced membrane depolarization, but instead caused hyperpolarization (Figure 4). Although membrane hyperpolarization has been described for other flavonoids,^57^ membrane permeabilization and hyperpolarization is a phenomenon rarely described in the literature. In fact, and unlike what we observed for the RES derivatives, hyperpolarized cells showed no uptake of propidium iodide.^57^ This result suggests a dual activity of the RES derivatives tested, which is similar to that observed in some compounds such as the human skin fatty acid cis-6-hexadecenoic acid^58^ and 3-*p*-*trans*-coumaroyl-2-hydroxyquinic acid.^59^ Altogether, these data suggest that the RES derivatives could act as ionophores inducing the membrane hyperpolarization by one side and, at the same time, perturbing membrane integrity during the interaction by forming pores and finally lysing the cells.

### RES Derivatives Impair Bacterial Respiration and Intracellular ATP Levels

**Figure 5:**
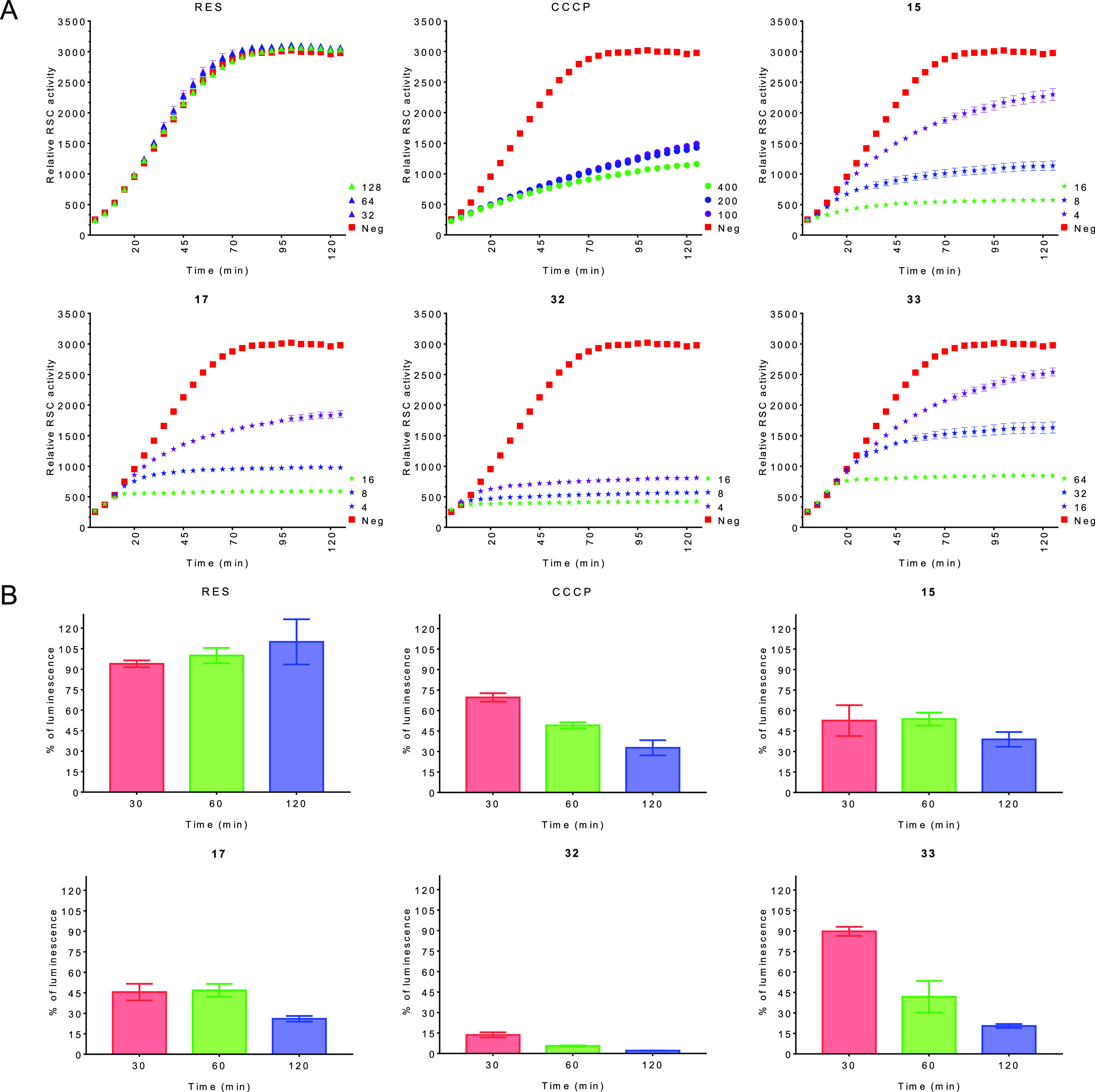
(A) Activity of the respiratory chain for RES (resveratrol), CCCP (carbonyl cyanide *m*-chlorophenyl hydrazone), and compounds **15**, **17**, **32**, and **33**, measured by reduction of resazurin (RSC) to resofurin ± SEM. The concentration of the compounds is expressed in μM. (B) Intracellular ATP levels expressed as % with respect to the negative control ± SEM. All the tests were performed in triplicate. Neg: negative control (nontreated cells).

Cell membrane dysfunction, as evidenced by the cell membrane hyperpolarization, impairs bacterial critical processes such as the respiratory chain and decreases ATP intracellular concentration.^60,61^ The effect on the respiratory chain was measured by the reduction of resazurin to resofurin, which is commonly used to determine respiratory dysfunction.^62,63^ As we can see in Figure 5A, incubation with RES derivatives diminished the reductive capacity of *B. cereus* ATC10987 cells, which is indicative of strong inhibition of the electron transport chain in a dose-related manner. For some derivatives, such as compound **32**, and also for some doses of the other three compounds, the effect was comparable to or even higher than that of the proton ionophore decoupler of the electron transport chain CCCP (carbonyl cyanide *m*-chlorophenyl hydrazone). No effect was observed for RES at the tested concentrations. Thus, the data suggest that RES derivatives could affect both the maintenance and generation of the proton motive force.

Finally, we analyzed if the ATP production could also be impaired by the RES derivatives. We measured the intracellular ATP using BacTiter-Glo microbial cell viability assay kit (Promega). The bacteria were incubated for 120 min with 32 μM of the compounds (100 μM for CCCP) at 37 °C, and we measured the ATP levels at 30, 60, and 120 min. As it can be seen in Figure 5B, RES did not statistically affect the ATP levels with respect to the control, whereas a time reduction of these levels was observed for the positive control CCCP. A similar outcome was observed for the RES derivatives, especially for compound **32**, which reduced dramatically the ATP levels inside the cells. Overall, the results suggest that the RES derivatives can produce an uncoupling of the proton gradient established during the normal electron transfer chain process given the membrane hyperpolarization and respiration impairment. Besides, RES has been proposed to inhibit ATP synthase activity reducing the intracellular levels of ATP,^21^ so we may not dismiss a stronger effect of the designed derivatives on this enzyme. Low ATP intracellular levels and membrane hyperpolarization are related also to membrane permeabilization,^61^ inducing the lysis of the cell and the bactericidal effect observed. With respect to the electron transfer chain, no effect of RES has been proved. In fact, it could be used as a coenzyme Q precursor, a lipid essential for electron and proton transport in the respiration process.^64^ These data suggest a completely alternative mechanism of action for the designed RES derivatives compared to the original RES molecule.

### Alkyl Sulfate RES Derivative **15** Shows a Synergistic Effect with Traditional Antibiotics

One of the most desirable characteristics of new antimicrobials is that they should not act antagonistically or generate cross-resistance with traditional antibiotics.^65^ The development of synergism that enhances the biocidal potential of the combination is currently one of the most promising research lines to fight multidrug-resistant bacteria. In fact, it is considered as a powerful alternative to elongate the half-life of traditional antibiotics, since the booster potential of the combinations aims for the total eradication of the pathogenic bacteria, avoiding the development of bacterial resistance.^12^ For this reason, we have explored the potential of 3-butyl-4′,5-disulfate resveratrol **15** and monosilyl derivative **17**, in combination with 24 traditional antibiotics against *E. faecium* LMG16003 and *S. aureus* LMG82224. These organisms are listed in the priority list of the WHO for which new drugs and/or alternative therapeutic treatments are urgently needed. Among the tested antibiotics, no antagonism was observed, and only a few drugs were able to show synergism. In the case of the silyl RES derivative **17**, an additive effect was mainly observed, while the alkyl sulfate derivative **15** showed strong synergistic effects. For the six best antibiotics, the reduction in the MIC values and the fractional inhibitory concentration index (FICI) calculation are listed in Table 4.

**Table 4:** MIC Reduction for Combinations of Antibiotics and Two Sub-MIC Concentrations of the RES Derivatives^a^

	*E. faecium* LMG16003								*S. aureus* LMG8224							
	Ab	RES	**15**			**17**			Ab	RES	**15**			**17**		
CT→		8	4	8	FICI	2	4	FICI		4	2	4	FICI	2	4	FICI
Ami	>32	-	-	-	-	-	-	-	8 ± 0	8 ± 0	2 ± 0	0.5 ± 0	0.31	3.3 ± 0.5	0.8 ± 0.1	0.60
Bac	>32	>32	16 ± 0	0.3 ± 0.2	0.27*	32 ± 0	16 ± 0	0.75*	>32	>32	26.6 ± 3.7	4 ± 0	0.31*	10.6 ± 1.8	6.6 ± 0.9	0.41*
Gen	32 ± 0	32 ± 0	13.3 ± 1.8	5.3 ± 0.9	0.41	13.3 ± 1.8	6.6 ± 0.9	0.66	1 ± 0	1 ± 0	0.5 ± 0	0.1 ± 0	0.37	0.3 ± 0	0.1 ± 0	0.58
Kan	>32	-	-	-	-	-	32 ± 0	1*	4 ± 0	4 ± 0	2 ± 0	0.6 ± 0.1	0.41	2 ± 0	1 ± 0	0.75
Pol	>32	>32	21.3 ± 3.7	1.8 ± 0.2	0.27*	-	-	-	>32	>32	8 ± 0	1 ± 0	0.25*	13.3 ± 1.8	6.6 ± 0.9	0.45*
Str	32 ± 0	-	-	-	-	-	-	-	16 ± 0	16 ± 0	-	-	-	8 ± 0	4 ± 0	0.75

a Concentrations are expressed in μM. Ab, antibiotic alone. CT, concentration of the compounds used in the synergism. Ami, Bac, Gen, Kan, Pol, and Str correspond to the antibiotics amikacin, bacitracin, gentamicin, kanamycin, polymyxin B, and streptomycin. FICI was calculated and interpreted as described by EUCAST.^66^ For scores ≤0.5 synergism, <0.5 additive effect, ≤1 and >1 indifferent, ≤2 and >2 antagonistic. - means not active under the conditions examined. * means relevant FICI values.

The reductions in MIC values observed for the combinations of **15** and **17** with aminoglycosides (amikacin, gentamycin, kanamycin, and streptomycin), especially against *S. aureus*, were quite remarkable since these antibiotics are not prone to act synergistically with other drugs.^50^ This effect is surprising since it is widely accepted that membrane voltage potentiates aminoglycoside activity^67^ and the RES derivatives tested disrupt it. However, it has been recently described that, in the absence of voltage, aminoglycosides enter cells and exert bacteriostatic effects by inhibiting translation.^68^ After that, cell killing is instantaneous upon repolarization. Membrane hyperpolarization and alteration in the ATP levels are two parameters observed for the compounds that are directly involved in such an effect.^68^ It has been described that RES also enhances the efficacy of aminoglycosides against *S. aureus*, which has been related to ATP synthase inhibition.^21^ In the case of RES, these effects were additive and obtained at concentrations 0.5× the MIC (about 560 μM), a concentration much higher than that observed for the alkyl RES derivative examined.^21^

It was also quite remarkable to observe the sensitization of polymyxin B produced by compounds **15** and **17**. Polymyxin B is a Gram-negative outer-membrane disruptive antibiotic that is almost inactive against Gram-positive bacteria. It has been previously observed that RES enhanced the antimicrobial effect of polymyxin B on Gram-negative bacteria but, similarly to the synergism with aminoglycosides, is observed at high dosages.^69^ The observed synergy could be related to an enhancement of the alternative mechanism of action described for this antibiotic.^70^ It could also be due to the low intracellular ATP levels induced by the RES derivatives. ATP synthase inhibition has been related to the elimination of the intrinsic polymyxin resistance in *S. aureus* and also sensitizes this bacterium to cationic antimicrobial peptides. This mechanism of action could also be responsible for the synergism observed for the cationic bacitracin.^71,72^ Bacitracin is a topically used antibiotic because of its toxicity, but its use as an oral drug to fight vancomycin-resistant enterococci has been considered.^73^ These combinations provide a boost of the antimicrobial activity against both *E. faecium* and *S. aureus*, which would contribute to reducing the toxicity of bacitracin and increase its applicability using new routes of administration.

### Hemolytic Activity and Cytotoxicity of RES Derivatives

**Figure 6:**
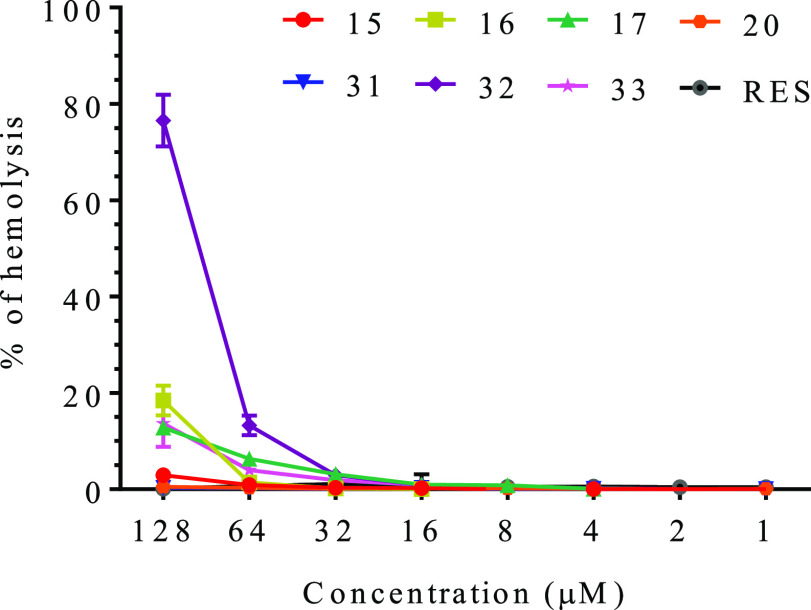
Hemolytic activity of resveratrol (RES) and selected resveratrol derivatives, compounds **15**, **16**, **17**, **20**, **31**, **32**, and **33**. Percent of hemolysis was calculated with respect to the positive control (Triton X-100) ± SEM.

Finally, we also examined the cytotoxicity and the hemolytic activity of some representative RES derivatives in human fibroblast MRC5 cells (Table 4) and human purified erythrocytes (Figure 6). Butyl sulfate RES derivative **15** was among the least toxic compounds (IC_50_ = 24.9 μM), yielding a 1.5- to 3-fold selectivity index (SI = IC_50_ MRC5/MIC) for several *B. cereus*, *S. aureus*, *E. faecalis*, and *C*. *tetani* strains. In the case of the silyl RES derivatives, compound **17** showed toxicity in the same range as **15**, with SI values between 2.0- and 3.8-fold for all *Bacillus*, *Enterococcus*, and *Staphylococcus* strains tested. However, the rest of the silyl RES derivatives turned out to be quite toxic with IC_50_ values between 1.8 and 2.8 μM (compounds **16**, **32**, and **33**) or less toxic but with no antibacterial activity (compounds **20** and **31**).

The tested compounds showed low hemolytic activity except at the highest concentration tested (128 μM). In this case, the derivative **32** was able to lyse 76% of the cells (Figure 6), while the derivatives **16**, **17**, and **33** lysed only 18.4%, 12.7%, and 13.6% of the red cells, respectively. No lysis was observed for RES and **15**. Overall and except for **32**, the lysis was below 5% for all the tested derivatives at the second highest concentration tested (64 μM, Figure 6). These data suggest that, in principle, the toxicity observed by the RES derivatives against eukaryotic cells may be caused by specific pathways non-membrane-damage-related as observed before for other stilbene-based resveratrol analogues.^74^

**Table 5:** Cellular Cytotoxicity for MRC5 Cells after 72 h Incubation Reported as IC_50_ Values (μM) Using MTT to Assess Cellular Metabolism^a^

	compound							
	RES	**15**	**16**	**17**	**20**	**31**	**32**	**33**
IC_50_ value	56.6 ± 3.2	24.9 ± 4.0	2.7 ± 0.3	15.5 ± 1.3	21.3 ± 5.9	32.1 ± 0.9	1.8 ± 0.1	2.8 ± 0.2

a Values expressed as mean ± standard error (*n* = 4–8).

In conclusion, among the potential health benefits provided by RES, its possible use as an effective antimicrobial is secondary and marginal since the high doses required prevents its implementation in the clinic. However, RES constitutes an excellent scaffold, as a starting point to design new compounds with increased antimicrobial activities in the range of traditional antibiotics. We have investigated the antimicrobial activity of a wide range of RES derivatives (glucosyl-acyl, alkyl, alkyl-sulfate, silyl, and silyl-acyl), observing good antimicrobial activity for specific compounds with some of these modifications, especially in the case of alkyl-sulfate RES and silyl RES derivatives. On the basis of these results, we designed and examined a set of new molecules combining the best chemical groups on RES, as well as newly designed silyl RES derivatives. We confirmed that not only the chemical group attached to the stilbene core but also the position of the group inside the molecule is important for the activity. Finally, we have investigated the mechanism of action for the best RES derivatives. We have observed a bactericidal mode of action and activity at the membrane level, where an unusual mode of action seems to be displayed involving membrane permeabilization and hyperpolarization, similarly to ionophore compounds. As a result of this activity, both the respiration chain and the ATP synthesis are impaired, which enhances the antimicrobial activity of these drugs. This observed mechanism of action is completely different from the one observed previously for RES and suggests a new route to kill Gram-positive bacteria. Activity against Gram-negative strains is low, since the outer membrane prevents these molecules from reaching the inner membrane in Gram-negative bacteria. Finally, we have discarded cross-resistance with traditional antibiotics. We also observed a synergistic activity with aminoglycosides and cationic antimicrobial peptide antibiotics against Gram-negative pathogens. However, preliminary toxicity data suggest a possible toxicity of these molecules against some cell lines, which justifies further efforts into optimizing the druggability of these compounds and performing *in vivo* studies.

## Experimental Section

### General Experimental Procedures

All solvents and chemicals were obtained from chemical suppliers and used as purchased without further purification. RES (**1**) was obtained from Sigma-Aldrich. All reactions were monitored by TLC on F_254_ precoated silica gel 60 plates (Merck) and detected by heating after staining with H_2_SO_4_/EtOH (1:9, v/v) or Mostain (500 mL of 10% H_2_SO_4_, 25 g of (NH_4_)_6_Mo_7_O_24_·4H_2_O, 1 g of Ce(SO_4_)_2_·4H_2_O). Products were purified by flash chromatography with silica gel 60 (70–230 mesh). Eluents are indicated for each particular case. NMR spectra were recorded on Bruker Advance 400 or 500 MHz NMR spectrometers at room temperature for solutions in CDCl_3_ or MeOH-*d*_4_. Chemical shifts are referred to the partially deuterated residual solvent signal. Two-dimensional experiments (COSY, TOCSY, ROESY, and HMQC) were carried out when necessary to assign the new compounds. Chemical shifts are expressed in ppm. High-resolution mass spectra (HRMS) were obtained on an ESI/quadrupole mass spectrometer (Waters, Acquity H Class).

### Synthesis of Compounds

Compounds **2**–**6**,^40^**7**,^41^**8**,^45^**9**–**12**,^47,48^**13**–**15**,^42^**16**–**25**,^43^**26**–**28**,^41^**29** and **30**,^46^ and **31**–**33**^43^ were synthesized and fully characterized according to the literature.

#### General Procedure for Silylation

In a round-bottom flask under agitation resveratrol (1 equiv) and imidazole (2.5 equiv) were suspended in dimethylformamide (DMF) (3 mL/mmol of resveratrol) and cooled to 0 °C. The corresponding silyl chloride (1.4–1.55 equiv) was added dropwise in a two-step process while stirring, half of the amount at *t* = 0 h and the rest at *t* = 3 h. The reaction was stirred for another 6 h at room temperature The reaction mixture was filtered, diluted with H_2_O, and extracted with EtOAc (3 × 50 mL). The combined organic layers were dried with MgSO_4_ and then filtered, concentrated, and added to a silica gel column eluting with gradient concentrations of hexane/EtOAc.

#### General Procedure for Sulfation

In a 25 mL round-bottomed flask the silyl resveratrol derivative to be sulfated (1 equiv) and SO_3_·Et_3_N complex (10 equiv) were dissolved in anhydrous MeCN (ca. 10 mL). Et_3_N (20 equiv) was then added. The reaction took place under agitation in a microwave (150 W, 60 °C, 1 h). After completion, the mixture was filtered and concentrated. The product was resuspended in i-PrOH, filtered, and purified in a silica gel column using hexane/EtOAc mixtures as eluents.

#### 3,5-Disulfate-4′-*tert*-butyldimethylsilyl resveratrol (**34**):

Yield = 75%; ^1^H NMR (400 MHz, MeOH-*d*_4_) δ 7.47 (2H, d, *J* = 8.5 Hz), 7.35 (2H, d, *J* = 1.8 Hz), 7.20–7.12 (3H, m), 7.01 (1H, d, *J* = 16.3 Hz), 6.85 (2H, d, *J* = 8.4 Hz), 1.02 (9H, s, 3 × CH_3_), 0.24 (6H, s, 2 × Si-CH_3_); ^13^C NMR (101 MHz, MeOH-*d*_4_) δ 155.57, 153.15, 139.42, 130.64, 129.24, 127.59, 125.40, 119.97, 115.17, 113.31, 53.79, 24.76, 17.69, −5.68; TOF MS ES *m*/*z* 501.0721 [M – H]^−^ (calcd mass for C_20_H_24_O_9_S_2_Si, 501.0709).

#### 3,4′-Disulfate-5-triisopropylsilyl resveratrol (**35**):

Yield = 31%; ^1^H NMR (400 MHz, MeOH-*d*_4_) δ 7.38 (2H, dd, *J* = 8.6, 3.3 Hz), 6.97 (1H, dd, *J* = 16.2, 5.8 Hz), 6.88–6.74 (3H, m), 6.64–6.44 (2H, m), 6.27 (1H, t, *J* = 2.2 Hz), 1.33–1.25 (3H, m, 3 × Si-CH), 1.18–1.06 (18H, m, 6 × CH_3_); ^13^C NMR (126 MHz, MeOH-*d*_4_) δ 158.20, 157.13, 157.04, 156.97, 139.86, 128.92, 128.27, 127.54, 127.46, 125.43, 115.17, 115.16, 109.19, 105.80, 53.84, 48.24, 44.17, 17.07, 17.03, 16.86, 12.55, 12.27, 11.64; TOF MS ES^–^*m*/*z* 543.1166 [M – H]^−^ (calcd for C_23_H_31_O_9_S_2_Si^2–^, 543.1179).

#### 3-Sulfate-4′,5-diisopropylsilyl resveratrol (**36**):

Yield = 84%; ^1^H NMR (400 MHz, MeOH-*d*_4_) δ 7.68 (2H, ddd, *J* = 39.7, 5.7, 3.4 Hz), 7.42 (2H. t, *J* = 5.8 Hz), 6.99 (1H, d, *J* = 16.2 Hz), 6.94–6.84 (2H, m), 6.57 (1H, d, *J* = 26.4 Hz), 6.29 (1H, s), 1.19–1.07 (36H, m, 12 × CH_3_), 0.98–0.90 (6H, m, 6 × Si-CH); ^13^C NMR (101 MHz, MeOH-*d*_4_) δ 167.93, 132.20, 131.00, 130.61, 128.47, 127.88, 127.40, 119.72, 105.94, 67.70, 38.77, 30.23, 28.74, 23.55, 22.64, 17.04, 16.99, 13.01, 12.54, 12.51, 10.02; TOF MS ES– *m*/*z* 619.2942 [M – H]^−^ (calcd for C_32_H_52_O_6_SSi_2_, 619.2945).

### Bacterial Strains and Culture Conditions

*Acinetobacter baumannii* LMG1041, *Klebsiella aerogenes* LMG2094, *K. pneumoniae* LMG20218, *Enterobacter cloacae* LMG2783, *Escherichia coli* LMG8223, *Pseudomonas aeruginosa* LMG6395, and *Salmonella enterica* LMG7233 as well as the Gram-positive bacteria *Bacillus cereus* ATCC10987 and *B. cereus* ATCC14579 were routinely grown in LB medium, shaking at 37 °C. *Enterococcus faecalis* V583,^75^*E. faecalis* LMG8222, *E. faecalis* LMG16216, *E. faecium* LMG11423, *E. faecium* LMG16003, *Staphylococcus aureus* LMG8224, *S. aureus* LMG10147, and *S. aureus* LMG15975 were grown in M17 medium plus 0.5% glucose (GM17) at 37 °C. *C. botulinum* CECT551, *C. tetani* CECT4629, *C. perfringens* CECT376, and *Clostridioides difficile* CECT531 were grown in reinforced clostridium medium (RCM) at 37 °C in anaerobiosis in Coy Lab’s vinyl anaerobic chambers, which provide a strict anaerobic atmosphere of 0–5 ppm (ppm) using a palladium catalyst and hydrogen gas mix of 5%. Agar at 1.2% was added for solid media if necessary. LMG strains were obtained from the Belgian Coordinated Collections of Microorganisms, ATCC strains from the American Type Culture Collection, and the CECT from the Spanish Type Culture Collection.

### Determination of the Minimal Inhibitory Concentration (MIC) Test

Each resveratrol derivative was diluted in a 10 mM DMSO stock solution and assayed at concentrations ranging from 128 to 2 μM against target bacteria using the broth microdilution method according to the CLSI guideline.^76^ The resveratrol derivatives were serially diluted in cMHB (BD Difco) in 96-well microplates. Plates were then inoculated with the indicator bacteria at a final concentration of 5 × 10^5^ CFU/mL and incubated at 37 °C for 20 h. In the case of the anaerobic bacteria (*Clostridium* and *Clostridioides*), the test was performed following the recommendation of CLSI for anaerobes^77^ but using RCM medium instead of cMHB. The cultures were cultivated inside an anaerobic chamber at 37 °C for 24 h. All the tests were performed in triplicate.

### Time Killing Kinetic and Bactericidal/Bacteriostatic Effect Determination

For the killing kinetic, an actively growing culture was diluted to an OD_600_ in cMHB to get about 10^5^–10^6^ CFU/mL. After that, 2× the MIC of the compounds **15**, **17**, **32**, **33**, and RES were added and the cultures were grown at 37 °C for 24 h. At 1, 2, 4, 6, and 24 h a sample of each treated culture was removed and decimal-serially diluted for CFU/mL counting. To confirm bactericidal/bacteriostatic activity, the minimal bactericidal concentration was calculated. For that, a MIC test was performed at concentrations from 128 μM to 2 μM. After that, the bacteria were inoculated at 10% in a new cMHB medium and incubated at 37 °C for 24 h. The absence of growth in the same range as the MIC indicated bactericidal activity, while the presence of growth indicated the bacteriostatic effect. The tests were performed in triplicate.

### Outer Membrane Permeabilization Test

To check if the outer membrane acts as a permeability barrier that protects Gram-negative bacteria against the resveratrol derivatives, *E. coli* LMG8223 was permeabilized with the outer-membrane-disturbing peptide L-11. Briefly, a MIC test in cMHA was performed for the different resveratrol derivatives in the presence of 4 μM of this peptide, which forms pores in the outer membrane. After that, the bactericidal/bacteriostatic effect was also tested as described before. The test was performed in triplicate.

### Activity on the Bacterial Membrane

#### Effect of the Compounds on Actively Growing Bacteria

To test if the compounds act bacteriolytically, *B. cereus* ATTC10987 was used as a model. Briefly, the strain was inoculated at 2% in cMHB medium and incubated at 37 °C in an Infinite 200Pro incubator (TECAN) monitoring the OD_600_ every 5 min during 8 h. At the middle of the logarithmic growth phase, the compounds at three different concentrations (2×, 1×, and 0.5× MIC) were added. A clear reduction in the OD_600_ value was related to cell lysis. Gramicidin S at 16 and 8 μM was used as a positive control. The test was performed in triplicate.

#### Membrane Integrity Assay

*B. cereus* ATTC10987 was grown in LB with shaking at 37 °C for 18 h. After that, the cells were centrifuged and washed in 5 mM HEPES buffer (pH 7.4) plus 5 mM glucose (G-HEPES buffer) three times, adjusted to an OD_600_ of 0.5 in the same buffer plus 10 μg/mL of propidium iodide (Sigma-Aldrich), and incubated for 10 min at 37 °C. This is a nonfluorescent dye impermeable to the bacterial membranes, but, if the membranes are disturbed, the dye can enter by binding the DNA, providing a strong fluorescent signal. In a 96-well plate, the different compounds to be tested were plated at 2× the desired concentration (2×, 1×, and 0.5× the MIC) in G-HEPES buffer. Once the cells were saturated with the dye, they were mixed 1:1 with the resveratrol derivatives. The fluorescence was monitored at 535/615 nm every 5 min during 75 min in a Varioskan Flash incubator (Thermo Scientific) at 37 °C. Gramicidin S (Sigma-Aldrich) was used as a positive control. The experiment was performed in triplicate.

#### Membrane Potential Assay

*B. cereus* ATCC10987 was cultured at 37 °C in LB medium to an OD_600_ of 1. The cells were washed three times with G-HEPES buffer (pH 7.4) and suspended in the same buffer at a final OD_600_ of 0.5. 3,3-Dipropylthiadicarbocyanine iodide, DiSC3(5) (Sigma-Aldrich), was added to the cells to a final concentration of 2 μM, and the cells were incubated at 37 °C for 20 min to set the basal fluorescence. As before, the resveratrol derivatives to be tested were plated in a 96-well plate at 2× the desired concentration (2×, 1×, and 0.5× the MIC) in G-HEPES buffer. Once the cells were loaded with the dye, they were mixed 1:1 with the resveratrol derivatives and the fluorescence was monitored at 622/670 nm every 5 min during 75 min in a Varioskan Flash incubator (Thermo Scientific) at 37 °C. Gramicidin S (Sigma-Aldrich) was used as a positive control for the cell permeabilization, and nontreated cells and just medium with the dye as negative controls. The experiment was performed in triplicate.

### Activity of the Respiratory Chain

The effect of the resveratrol derivatives on the *B. cereus* ATCC10987 respiratory chain was measured by the reduction of resazurin to the fluorescent resorufin. The bacteria were cultured in LB medium at 37 °C to an OD_600_ of 1. Then, the cells were washed three times in cMHB and resuspended in this medium at a final OD_600_ of 0.2. Resazurin at 100 μg/mL was added to the cells that were distributed in a 96-well plate. The different resveratrol derivatives to be tested were added to the plate at the indicated concentrations, and the conversion of resazurin to resorufin was measured each 5 min at an excitation/emission of 550/590 nm during 125 min in a Varioskan Flash (Thermo Scientific). CCCP, which uncouples the respiratory chain from the proton gradient at 400, 200, and 100 μM, was used as a positive control. No treated cells and just MHB medium with resazurin were used as negative controls. All the tests were performed in triplicate.

### Intracellular ATP Level Quantification

The intracellular ATP levels were determined using a BacTiter-Glo microbial cell viability assay (Promega) kit. *B. cereus* ATCC10987 was cultured in LB medium at 37 °C until an OD_600_ of 1. Under these conditions, the cells were washed three times with G-HEPEs and suspended in cMHB at a final OD_600_ of 0.25. The cells were treated with the resveratrol derivatives at a 32 μM concentration at 37 °C, and after 30, 60, and 120 min 100 μL was removed, mixed with 100 μL of BacTiter-Glo reagent, and incubated for 5 min at room temperature. After that, the luminescence was measured in an Infinite 200Pro incubator (TECAN). CCCP (100 μM) was used as a positive control, and nontreated cells as negative control. The OD_600_ was determined before the ATP levels were measured, and the relative ATP levels were calculated by the OD_600_ with respect to the control. All the tests were performed in triplicate.

### Combined Action with Antibiotics

The combined activity of the best RES derivatives and antibiotics was tested. The MIC was calculated initially for amikacin, ampicillin, bacitracin, chloramphenicol, ciprofloxacin, coumermycin A1, erythromycin, gentamicin, kanamycin, linezolid, meropenem, minocycline, nalidixic acid, novobiocin, oxacillin, pentamidine, polymyxin B, rifampicin, streptomycin, tetracycline, trimethoprim, and vancomycin against *E. faecium* and *S. aureus*. Once the MIC was known, a second MIC value was measured in the presence of sub-MIC concentrations of the desired RES derivatives. For the synergistic antibiotics, a large checkerboard test was performed^78^ and the intensity of the combinatorial relation was determined by calculation of the FICI. FICI was calculated and interpreted as described by EUCAST.^66^ For scores ≤0.5 synergism, <0.5 additive effect, ≤1 and >1 indifferent, ≤2 and >2 antagonistic.

### Hemolytic Activity and Cytotoxicity

Human blood of healthy individuals was obtained from Sanquin (certified Dutch organization responsible for meeting the needs in healthcare for blood and blood products, https://www.sanquin.nl/). For the erythrocyte isolation, 10 mL of blood was centrifuged at 1000*g* at 4 °C for 10 min, and the yellow supernatant removed. After that, the cells were washed five times with a NaCl 0.9% solution (10 mL) in the same conditions and finally resuspended in the same volume of buffer (10 mL). In a 96-well plate, the resveratrol derivatives were added in a volume of 40 μL of NaCl 0.9%, and 160 μL of 10-fold diluted red cells was added to get a final concentration of the compounds ranging between 128 and 1 μM. Triton X-100 at 1% was used as positive lysis control. The mix was incubated at 37 °C for 1 h. After that, samples were centrifuged (1000*g*, at 4 °C for 10 min) to remove the intact erythrocytes, and the supernatant was transferred to a new 96-well plate. The release of hemoglobin absorbance was measured at OD_540_, and the % of hemolysis was calculated as [(*H*_A_ – *H*_0_)/(*H*_+_ – *H*_0_)] × 100 where *H*_A_ is the absorbance at OD_540_ of the samples, *H*_0_ is that for the negative control, and *H*_+_ is that for the positive control.

### Cellular Cytotoxicity on MRC5 Cells

The protocol was carried out as follows: MRC-5 cells (human lung fibroblasts) were grown in monolayer (37 °C, 5% CO_2_, and 100% humidity) in DMEM medium (1 g/L glucose), supplemented with 10% heat-inactivated fetal bovine serum, 2 mM l-glutamine, 100 U/mL penicillin, and 100 mg/mL streptomycin. Cells were cultured according to ATCC recommendations and were used for the experiments while in the exponential growth phase. Cytotoxicity was measured through the MTT assay (ThermoFisher Scientific). Briefly, 5 × 10^3^ MRC-5 cells per well were seeded in 96-well plates (100 μL/well) in the presence of increasing concentrations of compounds. After 72 h of incubation at 37 °C, 10 μL of MTT solution (5 mg/mL) was added to each well and cells were reincubated for 4 h at 37 °C. Then, cell media was aspirated, and 100 μL of DMSO was added to each well to solubilize the formazan crystals obtained. The plate was incubated at 37 °C for an extra hour, and then the absorbance at 570 nm was measured at the Infinite F200 plate reader (TECAN Austria, GmbH). The results are expressed as the concentration of compound that reduces cell growth by 50% versus untreated control cells (EC_50_) using GraphPad Prism 7 (GraphPad Software) to fit the data to a sigmoidal curve. Data are presented as the mean ± SEM of three independent measurements all conducted in triplicate conditions.

## Supporting Information Available

Spectra of compounds **34**–**36** (PDF)
