# Supplementary material for: Chemically Tuning Resveratrol for the Effective Killing of Gram-Positive Pathogens
Source: J Nat Prod. 2022 May 27;85(6):1459–73. doi: 10.1021/acs.jnatprod.1c01107 (PMC9237828; doi:10.1021/acs.jnatprod.1c01107)
Supplement: Supplementary file 1 — np1c01107_si_001.pdf [file np1c01107_si_001.pdf]

Supplementary Information

Chemically tuning resveratrol for the effective killing of Gram-positive pathogens

Rubén Cebrián*, Qian Li, Pablo Peñalver, Efres Belmonte-Reche, María Andrés-Bilbao, Ricardo Lucas, María Violante de-Paz, Oscar P. Kuipers* and Juan Carlos Morales*

Table of Contents.

Structure of compounds studied in this work — Pages S2-S4

^{1}H and ^{13}C-NMR spectra of compound **34** — Page S5

^{1}H and ^{13}C-NMR spectra of silyl sulfate resveratrol derivatives **35** — Page S6

^{1}H and ^{13}C-NMR spectra of silyl sulfate resveratrol derivatives **36** — Page S7

Stability studies of compounds **1**, **15**, **17**, **32** and **33** — Page S8

UPLC-MS conditions — Page S8

Supplementary Figure 1 — Page S9

Structure of compounds studied in this work

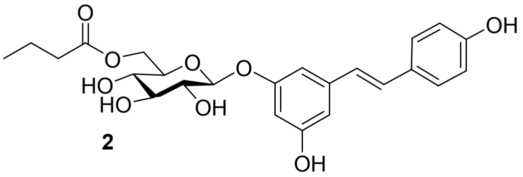

2

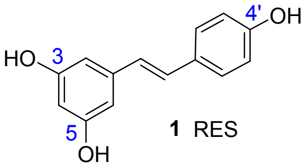

1 RES

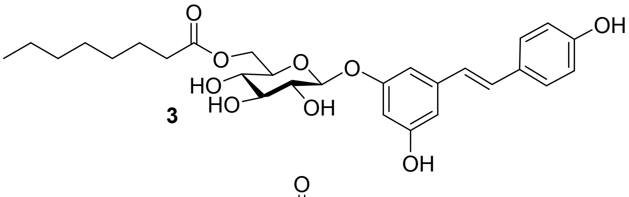

3

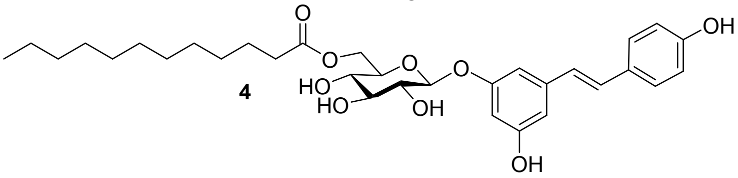

4

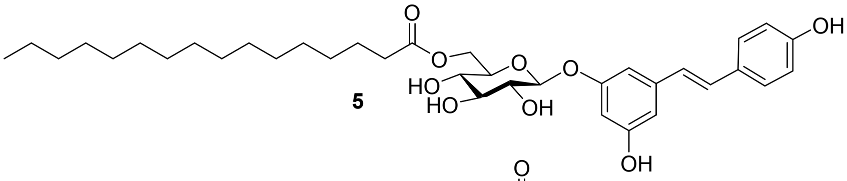

5

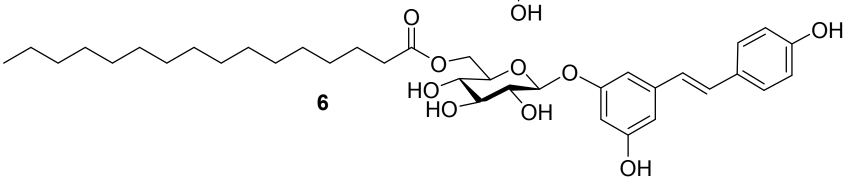

6

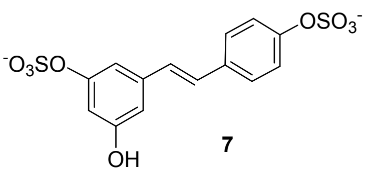

7

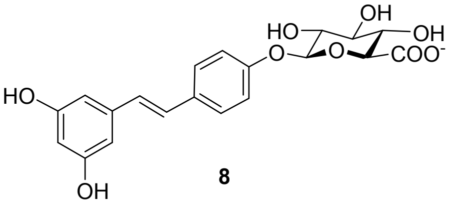

8

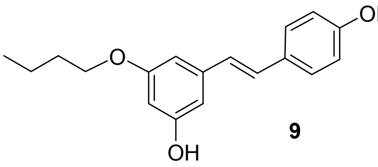

9

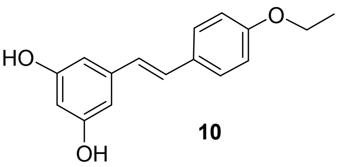

10

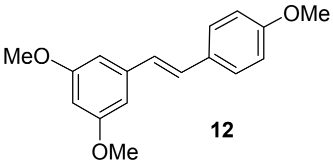

12

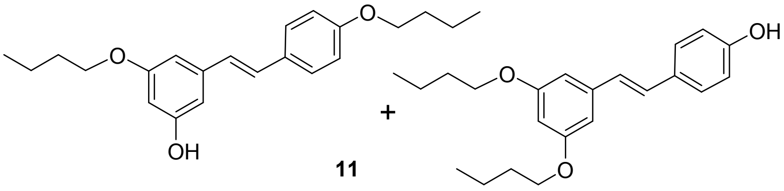

11

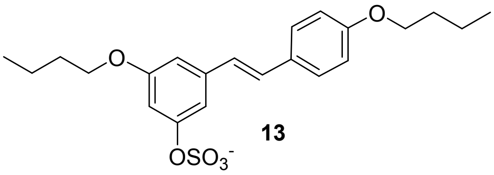

13

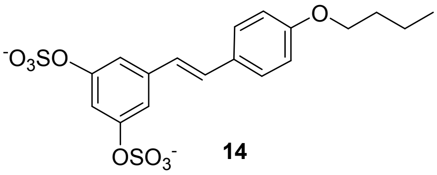

14

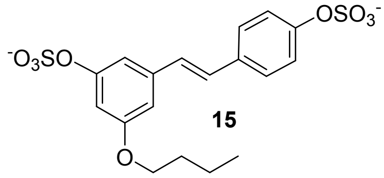

15

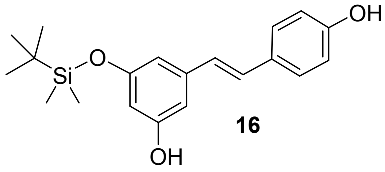

16

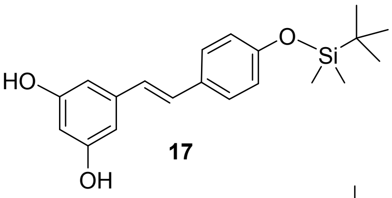

17

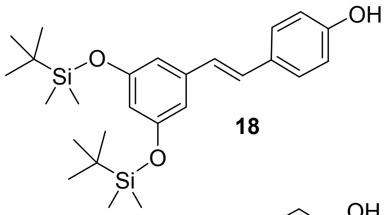

18

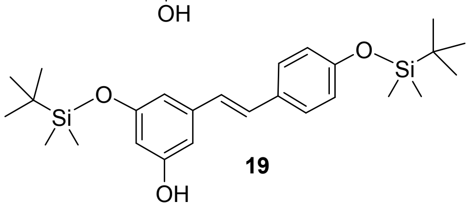

19

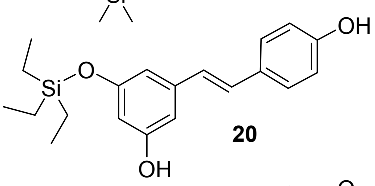

20

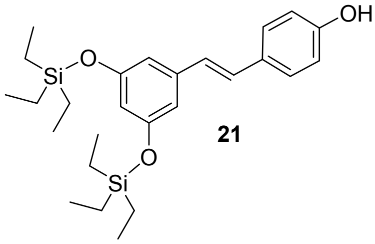

21

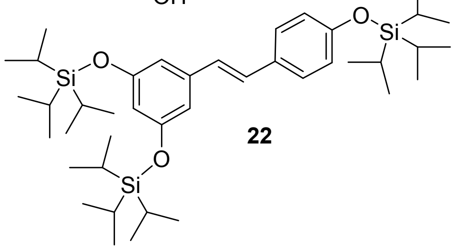

22

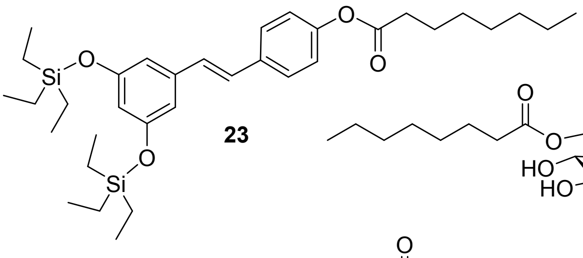

23

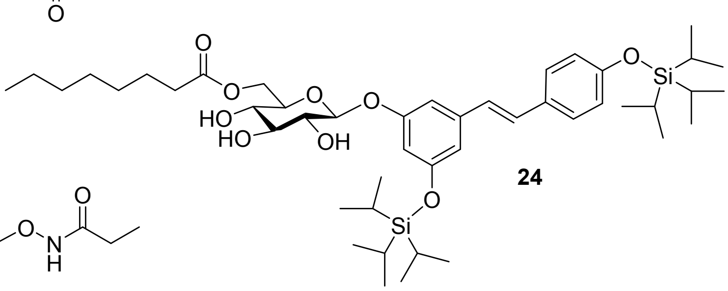

24

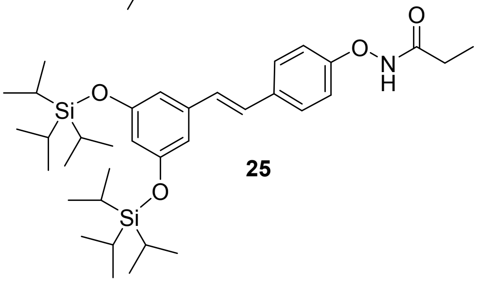

25

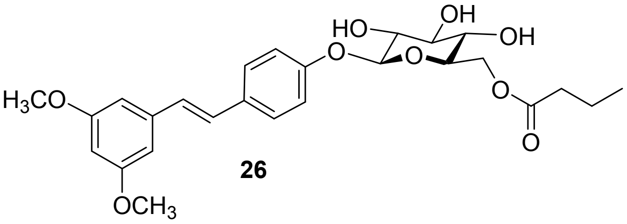

26

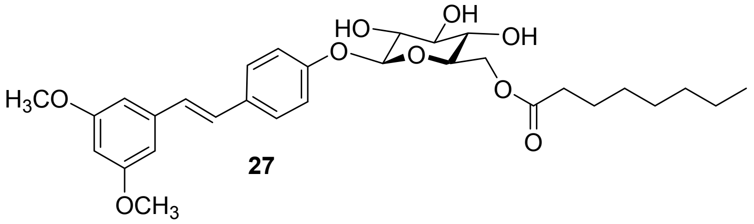

27

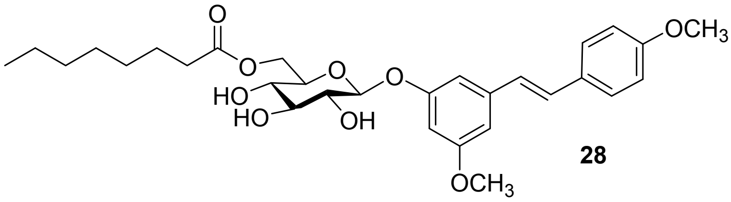

28

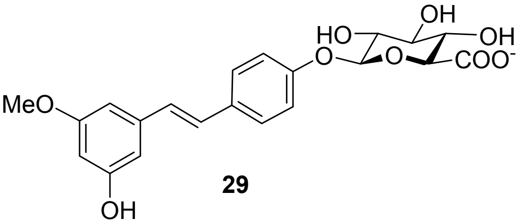

29

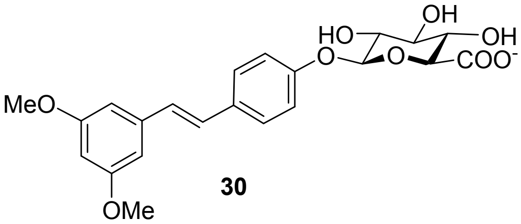

30

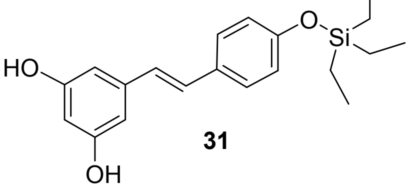

31

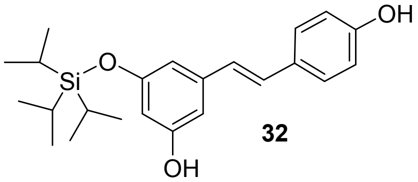

32

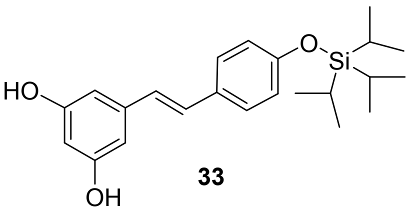

33

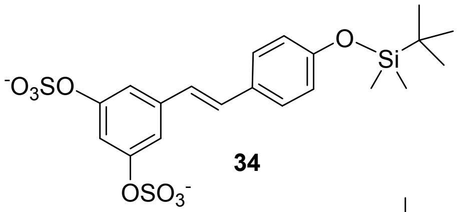

34

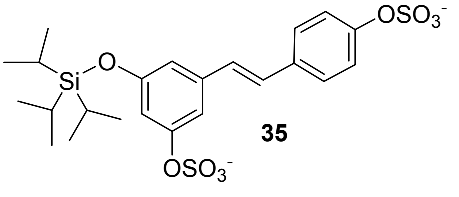

35

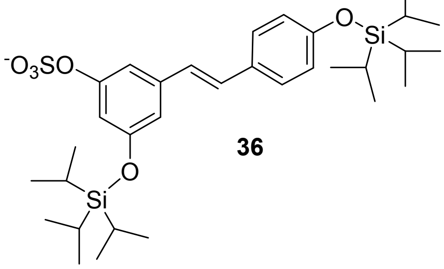

36

3,5-disulfate-4’-*tert*-butyldimethylsilyl resveratrol (**34**).

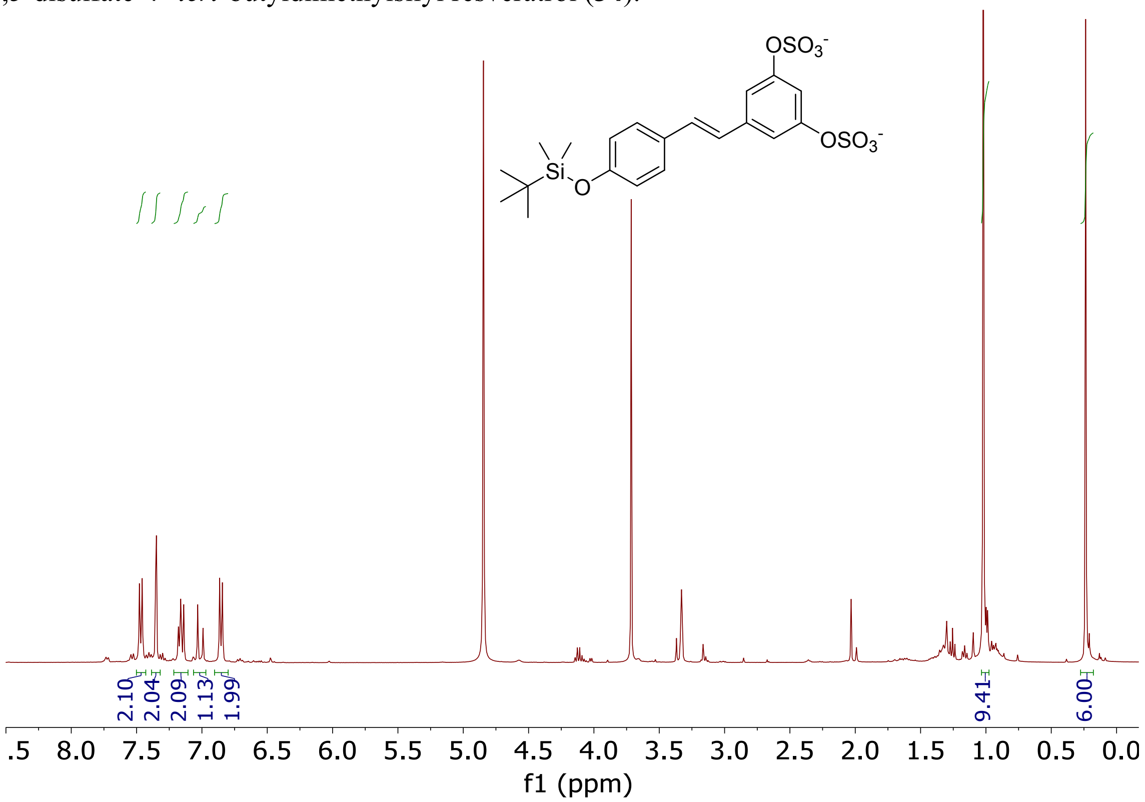

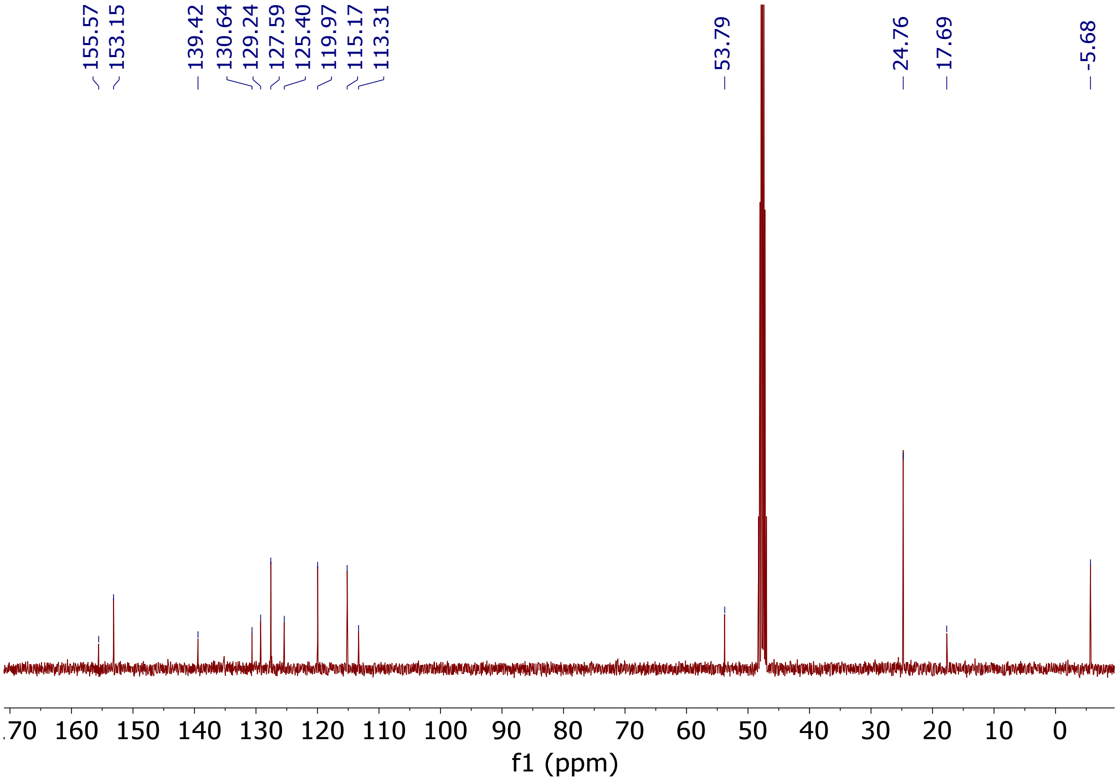

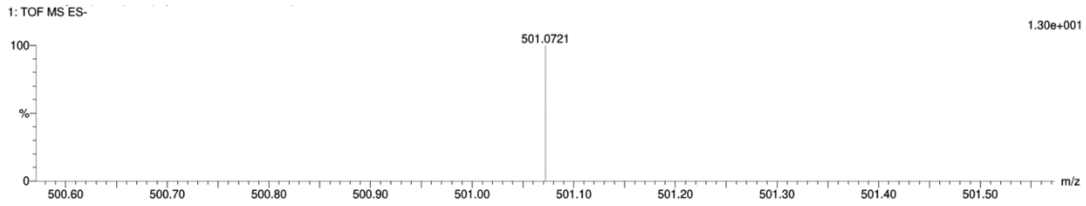

3,4’-disulfate-5-triisopropylsilyl resveratrol (**35**).

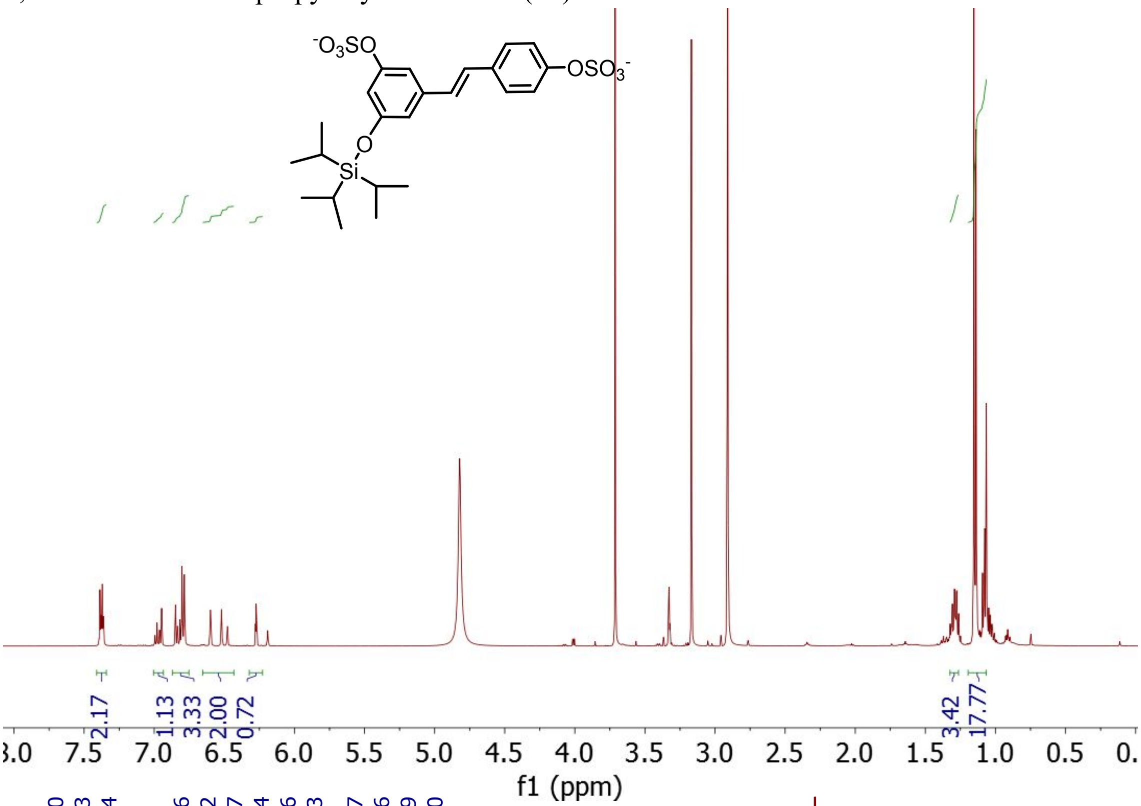

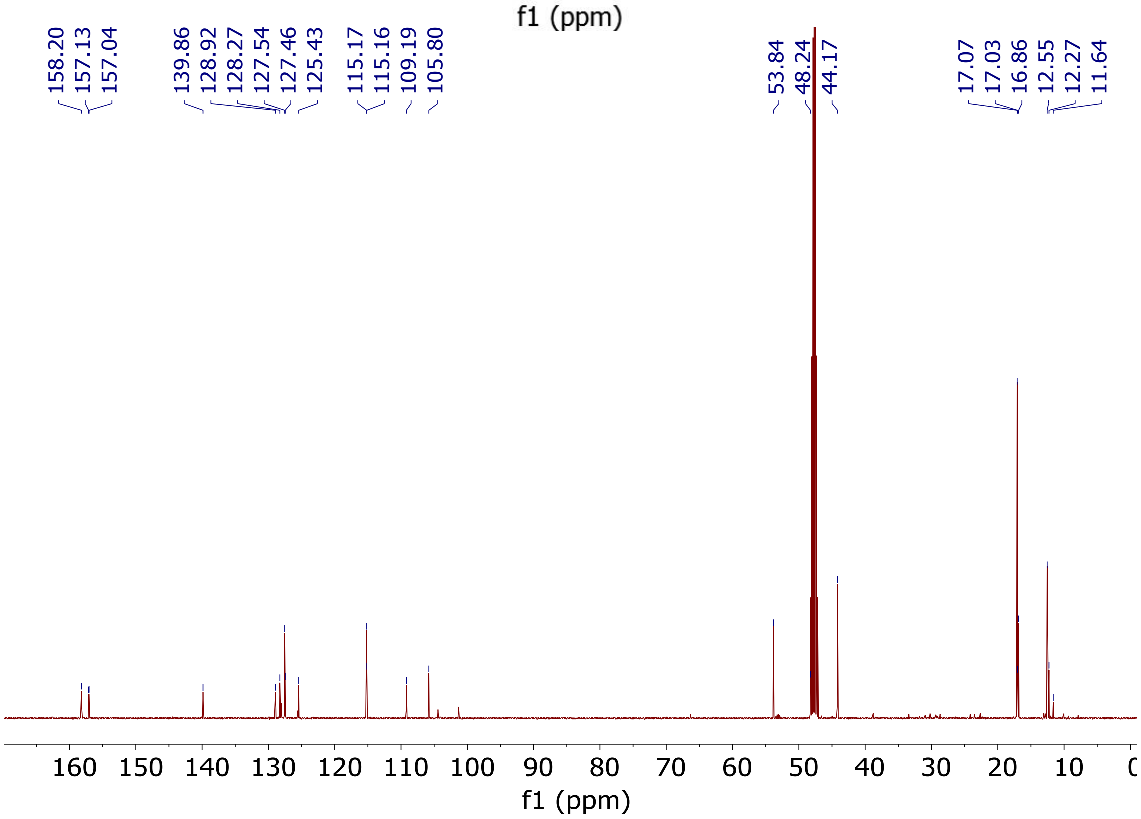

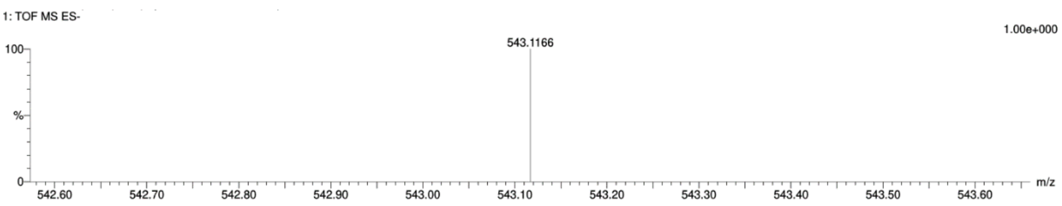

3-sulfate-4’,5-diisopropylsilyl resveratrol (**36**).

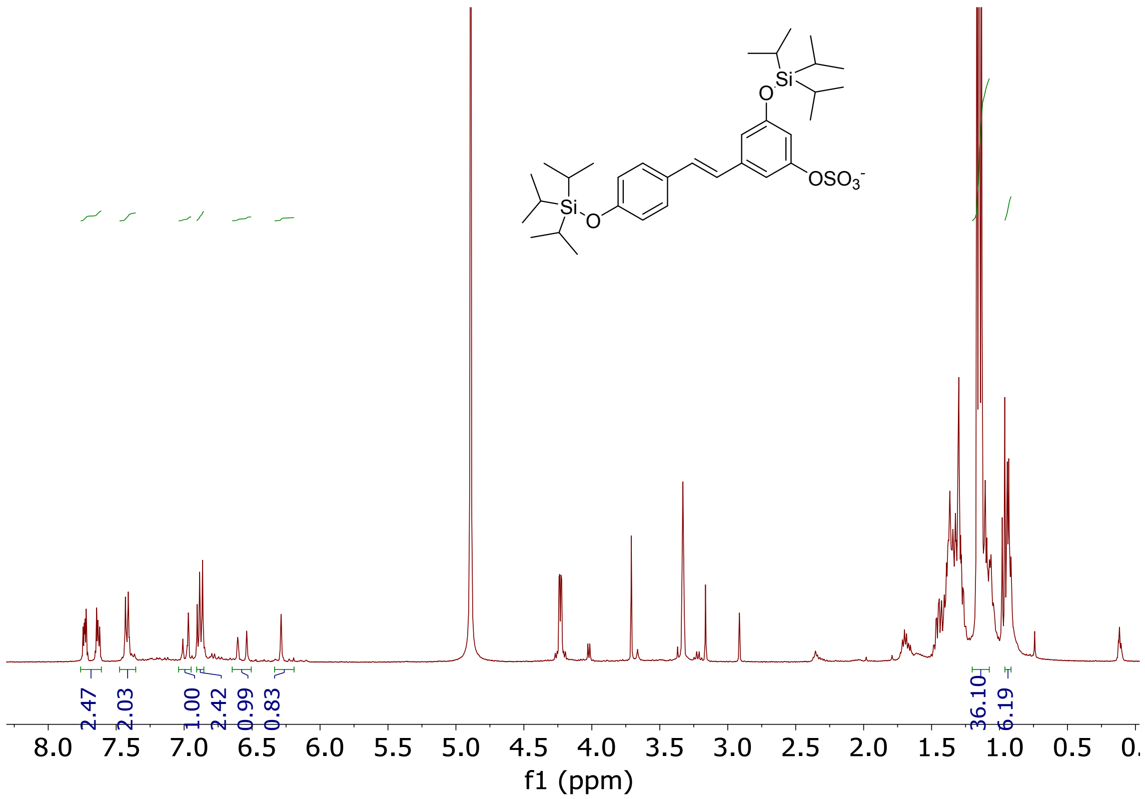

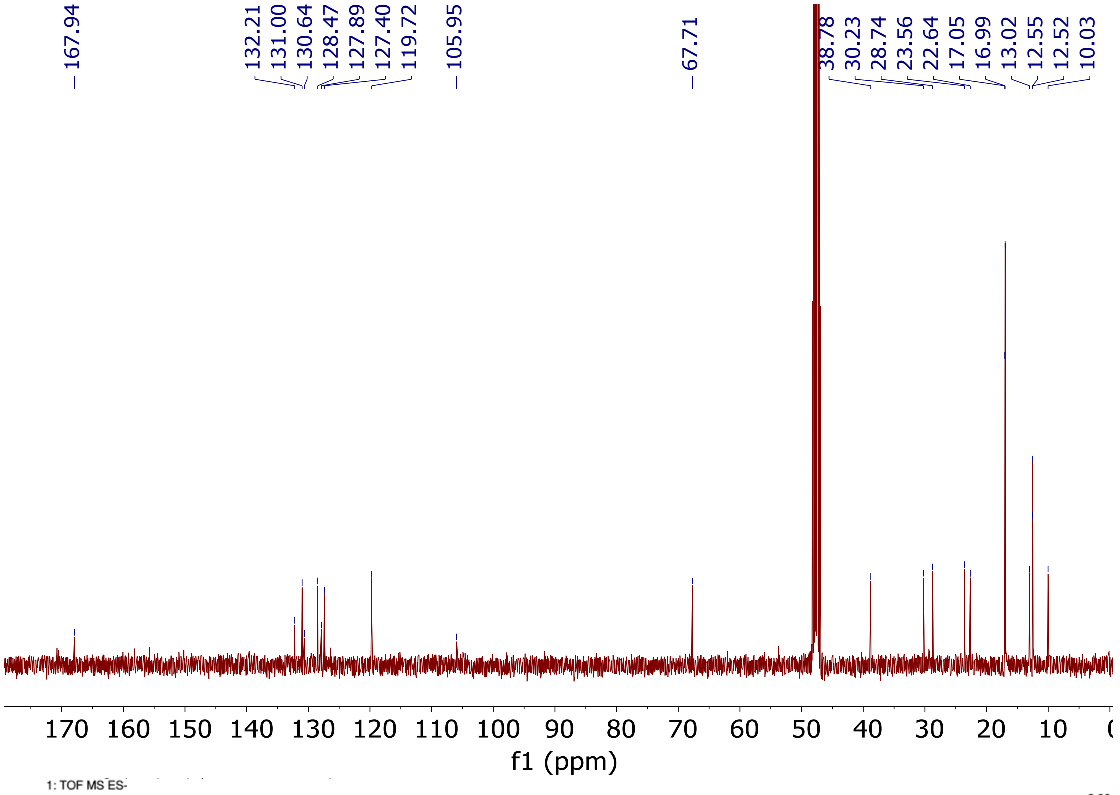

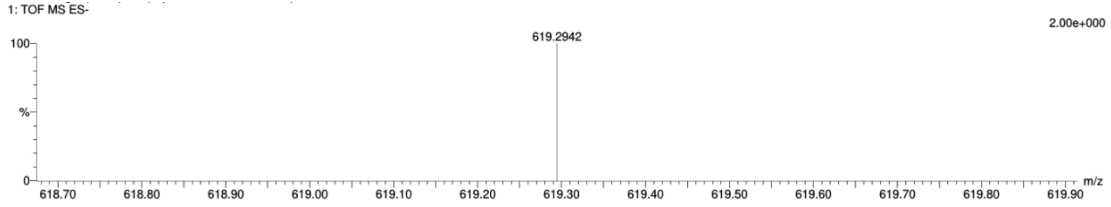

Stability studies of compounds 1, 15, 17, 32 and 33

Hydrolytic stability of resveratrol (**RES**, **1**) and the four more relevant compounds (**15**, **17**, **32**-**33**) was measured in the culture media used for MRC5 human fibroblast (DMEM) after 72 hours. Thus, compounds (100 μM) were incubated for 3 days in medium and the percentage of the remaining compound was calculated as the area under the curve of the chromatogram at t= 72 h versus t=0 h.

At the same time, hydrolytic stability of the same compounds, was measured in Muller-Hinton broth, the culture media used for bacterial growing at 24 h. To do so, 1mL of Muller-Hinton broth was incubated with 100 μM of the selected compounds 24h. Then after 1-minute centrifugation at 13 rpm, supernatants were collected and injected in the UPLC/MS equipment. The percentage of the remaining compound was calculated as the area under the curve of the chromatogram at t= 24 h versus t=0 h.

UPLC-MS conditions

Chromatographic separation was performed on a Acquity UPLC I Class System **(**Waters) using a binary gradient mobile phase consisting of H_2O + 0.1% formic acid (solvent A) and Acetonitrile (solvent B). The flow rate was 0.4 ml/min, the column (Waters UPLC HSS T3 2.1 x 100 mm, 1.8 µm) was maintained at 40ºC and the injected volume was 10 μl. Gradient conditions were as follows: 100% of A for 4 min, then 100 % of B for 4 more minutes and 1 more minute at the initial conditions (100% of A) to equilibrate the column.

The mass spectrometer (MS) was operated with electrospray ionization (ESI) in negative ion mode $[M-H]^-$ and the data were collected by selected ion recording mode (SIR).

A TUV- Detector with λ=254nm and 224 nm was used to record the PDA spectra. All compounds were quantified respect to its control (t= 0h) and results were expressed as percentage of compound remaining.

Supplementary Figure 1

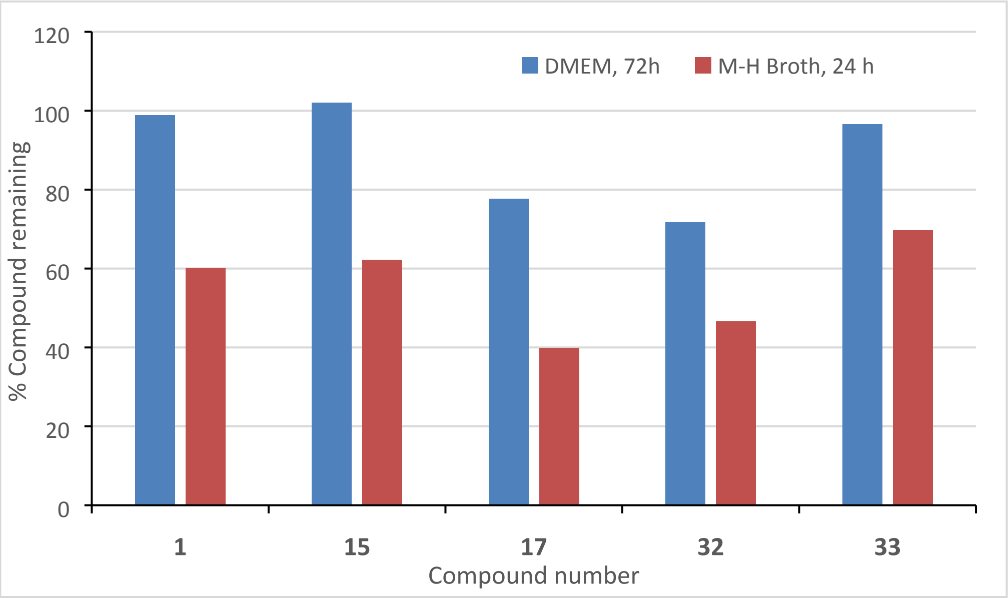
120
100
80
60
40
20
0
% Compound remaining
DMEM, 72h
M-H Broth, 24 h
1
15
17
32
33
Compound number
